# Supplementary material for: Positive rhizosphere priming accelerates carbon release from permafrost soils
Source: Nat Commun. 2025 Apr 15;16:3576. doi: 10.1038/s41467-025-58845-9 (PMC12000286; doi:10.1038/s41467-025-58845-9)
Supplement: Supplementary file 1 — Supplementary Information [file 41467_2025_58845_MOESM1_ESM.pdf]

# Supplementary information

## Positive rhizosphere priming accelerates carbon release from permafrost soils

Nina L. Friggens[1*], Gustaf Hugelius[2], Steven V. Kokelj[3], Julian B. Murton[4], Gareth K. Phoenix[5], Iain P. Hartley[1*]

[1]*Department of Geography, Faculty of Environment, Science and Economy, University of Exeter, EX4 4RJ Exeter, UK*

[2]*Department of Physical Geography, Stockholm University, Stockholm, Sweden*

[3]*Northwest Territories Geological Survey, Government of the Northwest Territories, Yellowknife, Northwest Territories, Canada*

[4]*Department of Geography, University of Sussex, Brighton, BN1 9SJ, UK*

[5]*Plants, Photosynthesis and Soil, School of Biosciences, University of Sheffield, Sheffield, S10 2TN, UK*

[*]Corresponding author: *n.lindstrom-friggens@exeter.ac.uk*

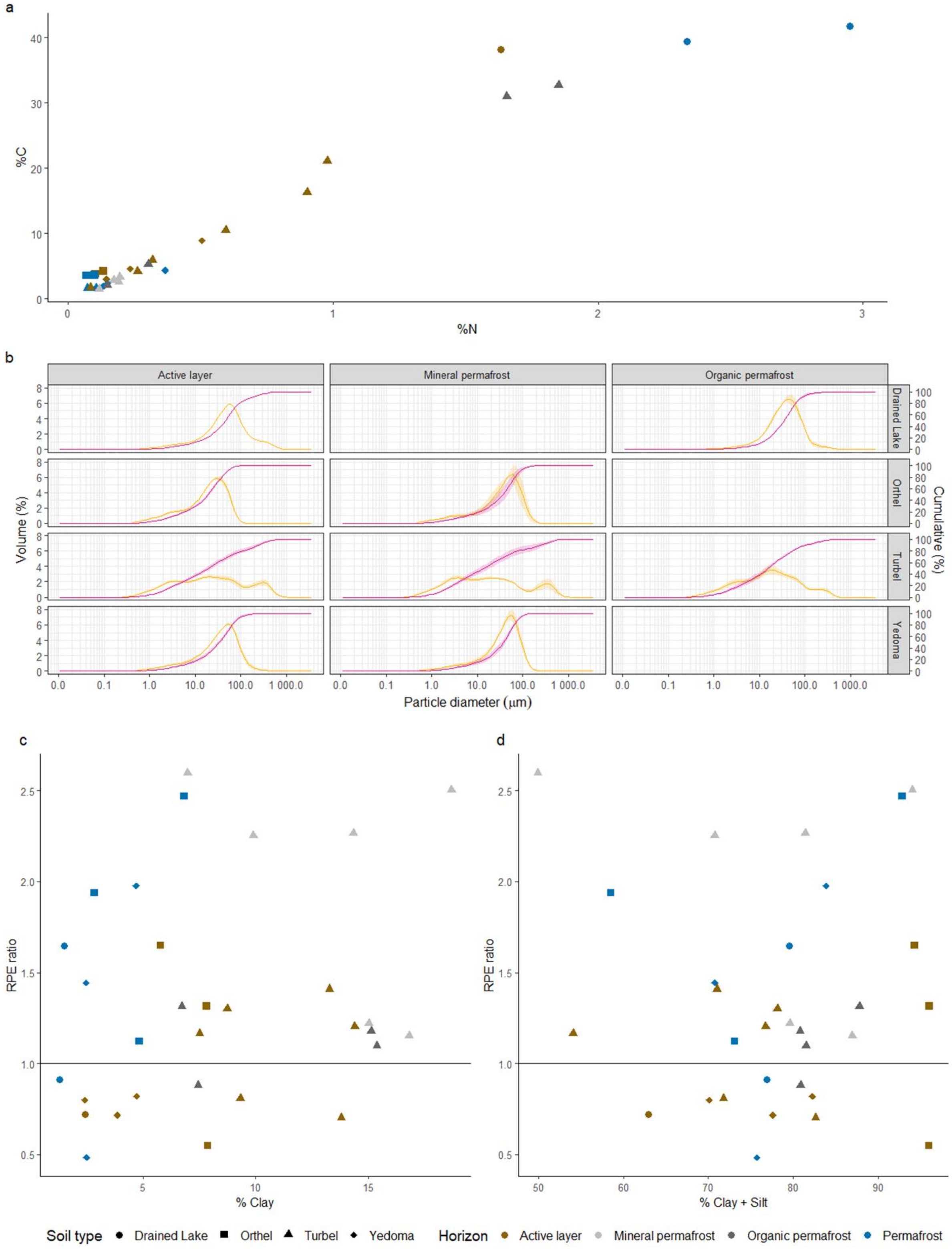


Supplementary Figure 1. **Soil sample C and N content as well as primary texture.** a) Distribution of C and N content across the samples included in the experiment. b) Soil particle-size distribution by volume (yellow) and cumulatively (maroon) averaged by soil type and horizon with standard error ribbons. c) RPE ratio relationship with soil % clay and d) RPE ratio relationship with soil % clay + sand. There were no significant relationships between RPE ratio and soil texture (c & d).

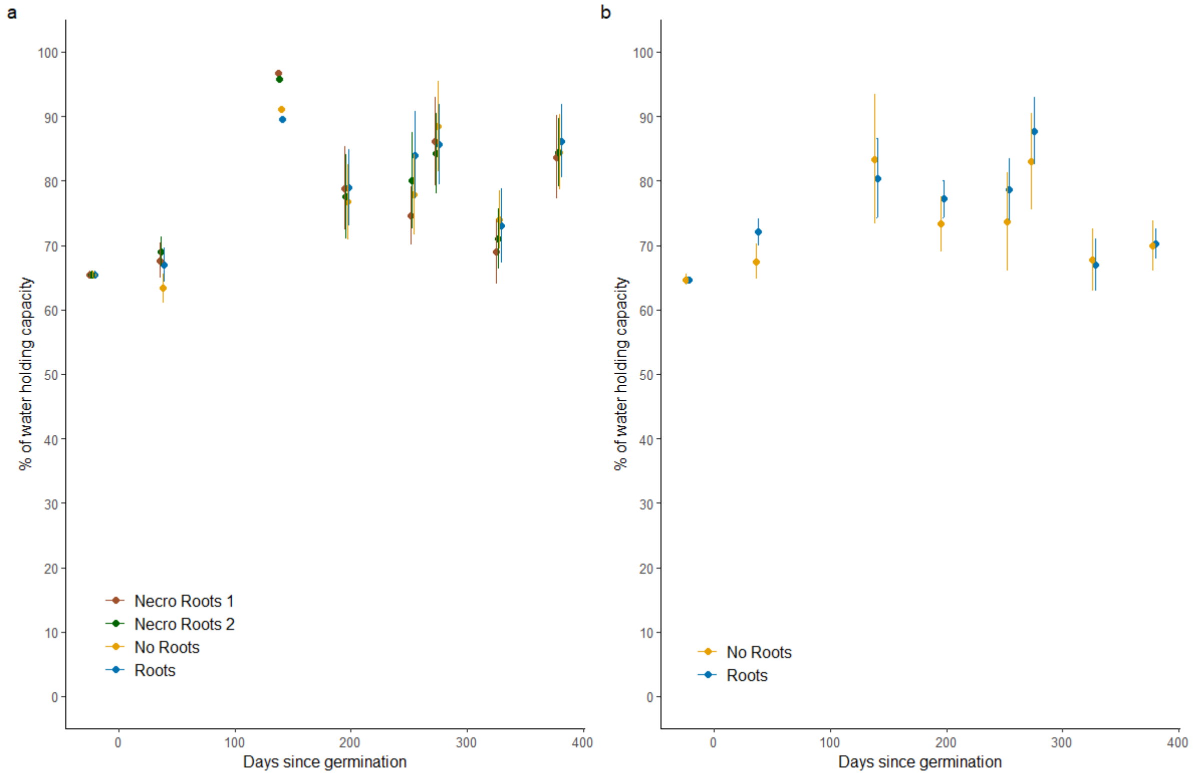


Supplementary Figure 2. **Soil moisture levels throughout the experiment.** Soil moisture throughout the experiment in mesocosms with root-exclusion mesh regime (a) and with additional nutrients (b). Error bars are standard error.

Whilst the soil moisture fluctuates over time and sits above the target of 65% of water holding capacity, there are no differences in soil moisture between soil with and without roots ($p > 0.5$) which could impact the RPE data.

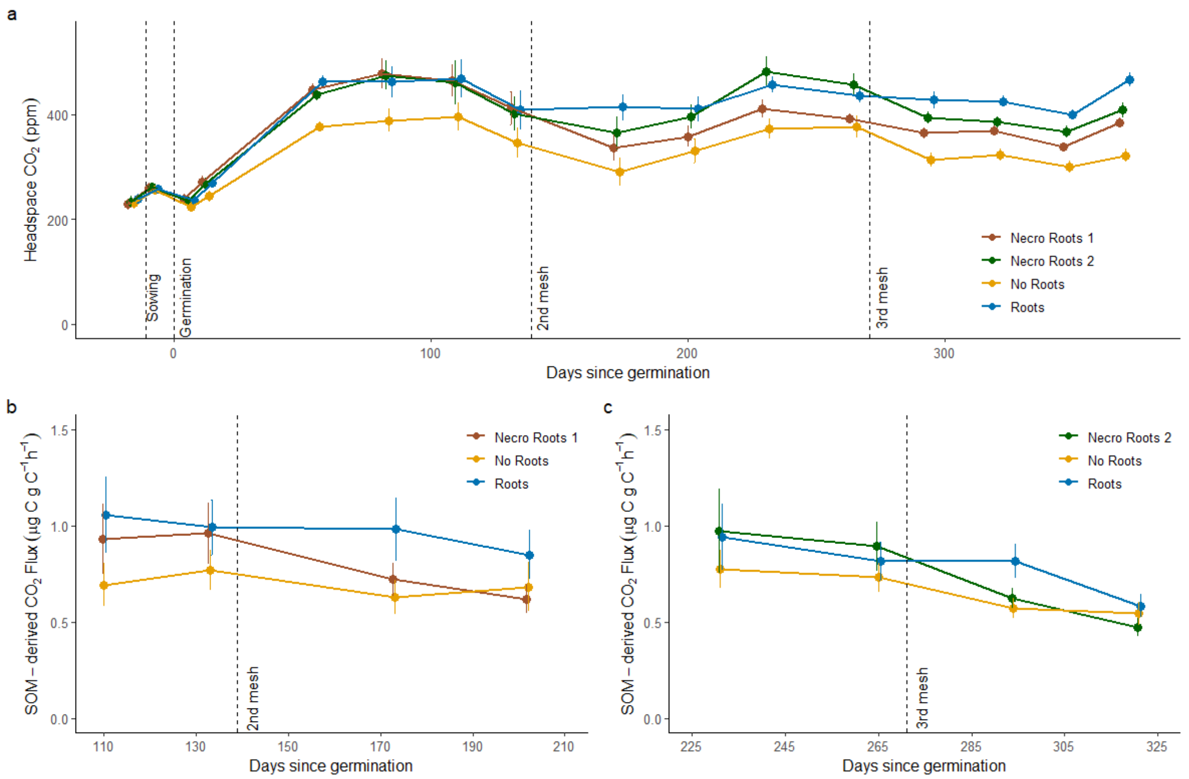


Supplementary Figure 3. **$CO_2$ concentrations and SOM-derived $CO_2$ fluxes throughout the experiment and at the time of mesh insertion**. Headspace $CO_2$ concentration in each side compartment treatment over time (a). ‘No roots’ was root-free control throughout. ‘Roots’ had root access throughout. ‘Necro roots 1’ had root access up until day 143 when roots were severed and further ingrowth prevented denoted by ‘2$^{nd}$ mesh’ line. ‘Necro roots 2’ had root access up until day 275 when roots were severed and further ingrowth prevented denoted by ‘1$^{st}$ mesh’ line. The flux of SOM-derived $CO_2$ from side compartments where roots were severed by the 2$^{nd}$ mesh (b) and 3$^{rd}$ mesh (c) decreased to the levels of the root-free control within one month of severing. Error bars are standard error.

The root-free side compartment had lower $CO_2$ concentrations in the headspace than rooted side compartments confirming that it remained an appropriate root-free control throughout the duration of the experiment (Supplementary Fig. 3a).

The headspace $CO_2$ concentration dropped in side compartments whenever roots where severed (Supplementary Fig. 3a; Necro roots 1 & 2). At the end of the experiment the rooted side compartment had the highest headspace $CO_2$ concentration, and the root-free side compartment had the lowest headspace $CO_2$ concentration with both necro root side compartments having intermediate headspace $CO_2$ concentrations showing a drop in root respiration following severing.

All three rooted side compartments (Roots, Necro roots 1 & 2) had higher SOM-derived $CO_2$ flux until the ‘2$^{nd}$ mesh’ was inserted when ‘Necro roots 1’ SOM-derived $CO_2$ flux drops to similar levels as the ‘No root’ control (Supplementary Fig. 3b). ‘Roots’ and ‘Necro roots 2’ remain high until the ‘3$^{rd}$ mesh’ was inserted when ‘Necro roots 2’ SOM-derived $CO_2$ flux drops to similar levels as the ‘No root’ control (Supplementary Fig. 3c). This suggests that the positive RPE, the difference in SOM-derived $CO_2$ flux between rooted and root-free soil, was primarily driven by fresh C inputs from live roots but not by the presence of root necromass.

## Permafrost soil samples

Supplementary Table 1. **Soil types and soil profile numbers.**

| **Soil type** | **Profile #** |
|---|---|
| Non-lake (alluvial) orthels | 8, 9, 10 |
| Drained thermokarst-lake sediments | 2, 3, 7 |
| Non-lake (till) turbels | 4, 5, 6, HUS, CRB_S |
| Yedoma (and derived) deposits | 12, 13, 14 |

| List of soil profiles | Vegetation and substrate |
|---|---|
| #2. Turbel-T-P1: | tussock tundra on mineral lake silt |
| #3. Orthel-T-P2: | erect-shrub tundra on organic lake silt |
| #4. Turbel-T-P3: | erect-shrub tundra on till |
| #5. Turbel-T-P4: | erect-shrub tundra on till |
| #6. Turbel-T-P5: | erect-shrub tundra on till |
| #7. Histel-T-P6: | erect-shrub tundra on organic lake silt |
| #8. Orthel-EC-P2: | old burn spruce forest on alluvium |
| #9. Orthel-EC-P3: | old spruce–lichen forest on alluvium |
| #10. Orthel-EC-P4: | old spruce forest on alluvium |
| #12. Orthel-K-P1: | spruce forest on loessal deposits |
| #13. Orthel-K-P2: | spruce forest on loessal deposits |
| #14. Orthel- K-P3: | shrubby vegetation on disturbed ground |
| CRB_S: | hummocky ericaceous shrub tundra |
| HUS: | hummocky ericaceous shrub tundra |

Supplementary Table 2. **Soil samples and contextual data.**

| Sample # | Latitude | Longitude | Soil type | Active-layer thickness (m) ± std dev | Active layer or permafrost (± cryoturbated) | Depth below ground surface (m) | Date collected | Short description & interpretation |
|---|---|---|---|---|---|---|---|---|
| 19-018-T-P1 | 69° 23' 22.5"N | 133° 27' 59.3"W | Turbel - Drained lake sediments | 0.38 ± 0.01 (n=10) | AL | 0.23–0.40 | 13/08/2019 | Organic layer (lake) |
| 19-019-T-P1 | 69° 23' 22.5"N | 133° 27' 59.3"W | Turbel - Drained lake sediments | 0.47 ± 0.02 (n=3) | Pc | 1.0–1.3 | 13/08/2019 | Organic rich, cryoturbated (lake) |
| 19-020-T-P1 | 69° 23' 22.5"N | 133° 27' 59.3"W | Turbel - Drained lake sediments | 0.47 ± 0.02 (n=3) | Pc | 1.5–2.0 | 13/08/2019 | Mineral soil, cryoturbated (lake) |
| 19-041-T-P2 | 69° 22' 53.6"N | 133° 17' 18.8"W | Orthel - Drained lake sediments | 0.38 ± 0.02 (n=10) | AL | 0.25–0.4 | 13/08/2019 | Black organic silt (lake) |
| 19-042-T-P2 | 69° 22' 53.6"N | 133° 17' 18.8"W | Orthel - Drained lake sediments | 0.30 ± 0.04 (n=10) | Pnc | 0.6–1.0 | 13/08/2019 | Black organic silt (lake) |
| 19-058-T-P3 | 69° 25' 04.3"N | 133° 00' 36.4"W | Turbel - Non-lake (till) | 0.64 ± 0.05 (n=5) | AL | 0.20–0.45 | 14/08/2019 | Silt–clay diamicton (till) |
| 19-059-T-P3 | 69° 25' 04.3"N | 133° 00' 36.4"W | Turbel - Non-lake (till) | 0.39 ± 0.05 (n=5) | Pc | 0.9–1.1 | 14/08/2019 | Silt–clay diamicton (till) |
| 19-074-T-P4 | 69° 24' 28.7"N | 133° 00' 17.2"W | Turbel - Non-lake (till) | 0.51 ± 0.05 (n=5) | AL | 0.3–0.45 | 14/08/2019 | Organic-rich |
| 19-075-T-P4 | 69° 24' 28.7"N | 133° 00' 17.2"W | Turbel - Non-lake (till) | 0.29 ± 0.05 (n=5) | Pc | 0.9–1.1 | 14/08/2019 | Peat |
| 19-076-T-P4 | 69° 24' 28.7"N | 133° 00' 17.2"W | Turbel - Non-lake (till) | 0.29 ± 0.05 (n=5) | Pnc | 1.3–1.5 | 14/08/2019 | Silt–clay diamicton (till) |
| 19-092-T-P5 | 69° 24' 02.9"N | 132° 59' 46.0"W | Turbel - Non-lake (till) | 0.72 ± 0.09 (n=5) | AL | 0.3–0.5 | 15/08/2019 | Silt–clay & organic bodies |
| 19-093-T-P5 | 69° 24' 02.9"N | 132° 59' 46.0"W | Turbel - Non-lake (till) | 0.48 ± 0.11 (n=5) | Pc | 0.95–1.10 | 15/08/2019 | Silt–clay diamicton (till) |
| 19-110-T-P6 | 69° 24' 05.6"N | 133° 02' 46.3"W | Histel - Drained lake sediments | 0.33 ± 0.03 (n=10) | Pnc | 0.7–0.9 | 16/08/2019 | Black organic silt (lake) |
| 19-126-EC-P2 | 68° 26' 11.9"N | 133° 49' 03.3"W | Orthel - Non-lake (alluvial) | 0.56 ± 0.06 (n=10) | AL | 0.35–0.55 | 19/08/2019 | Silty Bg–Bgw horizon |
| 19-127-EC-P2 | 68° 26' 11.9"N | 133° 49' 03.3"W | Orthel - Non-lake (alluvial) | 0.56 ± 0.06 (n=10) | Pnc | 1.2–1.4 | 19/08/2019 | Sand to silty sand (alluvial) |
| 19-143-EC-P3 | 68° 28' 18.3"N | 133° 51' 09.4"W | Orthel - Non-lake (alluvial) | 0.56 ± 0.10 (n=50) | AL | 0.3–0.50 | 19/08/2019 | Silty Bgs horizon |
| 19-144-EC-P3 | 68° 28' 18.3"N | 133° 51' 09.4"W | Orthel - Non-lake (alluvial) | 0.56 ± 0.10 (n=50) | Pnc | 0.9–1.1 | 19/08/2019 | Silt and sand (alluvial) |
| 19-160-EC-P4 | 68° 31' 17.0"N | 133° 51' 29.0"W | Orthel - Non-lake (alluvial) | 0.92 | AL | 0.3–0.5 | 20/08/2019 | Bg horizon |
| 19-161-EC-P4 | 68° 31' 17.0"N | 133° 51' 29.0"W | Orthel - Non-lake (alluvial) | 0.92 | Pnc | 1.1–1.3 | 20/08/2019 | C horizon, sandy silt (alluvium) |
| 19-194-K-P1 | 63° 50' 25.9"N | 139°06' 42.8"W | Orthel - Yedoma | 0.30 | AL | 18–32 | 23/08/2019 | Grey silt–sand (loessal) |
| 19-195-K-P1 | 63° 50' 25.9"N | 139°06' 42.8"W | Orthel - Yedoma | 0.30 | Pnc | 60–110 | 23/08/2019 | Dark grey, wet silt–sand (loessal) |
| 19-211-K-P2 | 63° 50' 30.9"N | 139°06' 53.1"W | Orthel - Yedoma | 0.30–0.45 | AL | 20–35 | 23/08/2019 | Dark grey, saturated silt (loessal) |
| 19-212-K-P2 | 63° 50' 30.9"N | 139°06' 53.1"W | Orthel - Yedoma | 0.30–0.46 | Pnc | 100–120 | 23/08/2019 | Dark grey, wet silt (loessal) |
| 19-228-K-P3 | 63° 55' 55.0"N | 138°54' 33.3"W | Orthel - Yedoma | 0.50 | AL | 20–50 | 24/08/2019 | Min–organic, heterogen (spoil) |

Supplementary Table 2. **Soil samples and contextual data continued.**

| Sample # | Latitude | Longitude | Soil type | Active-layer thickness (m) ± std dev | Active layer or permafrost (± cryoturbated) | Depth below ground surface (m) | Date collected | Short description & interpretation |
|---|---|---|---|---|---|---|---|---|
| 19-229-K-P3 | 63° 55' 55.0"N | 138°54' 33.3"W | Orthel - Yedoma | 0.50 | Pnc | 120–140 | 24/08/2019 | Grey silt & org patches (loessal) |
| CRB_S_1 | 67° 10' 52.0242" | 135° 43' 34.4166" | Turbel | | AL | Unknown | 25/08/2020 | blocky & mineral-rich; oxidized materials; mottled brown-grey; angular stones (shale/sandstone) |
| CRB_S_2 | 67° 10' 52.0242" | 135° 43' 34.4166" | Turbel | | Pc | Unknown | 25/08/2020 | lenticular ice lenses (0.2-0.5cm thick), dipping parallel to terrain surface (slope); angular shale/sandstone, up to 10cm in length |
| CRB_S_3 | 67° 10' 52.0242" | 135° 43' 34.4166" | Turbel | | Pc | Unknown | 25/08/2020 | irregular thick lenses; some massive structures with ice lenses to 10 cm thick; rounded pebbles |
| HUS_1 | 69° 0' 53.7978" | 133° 16' 45.951" | Turbel | 0.3 | AL | 0-30 | 23/09/2020 | top of active-layer from lower organic layer to depth of ~30cm |
| HUS_2 | 69° 0' 53.7978" | 133° 16' 45.951" | Turbel | | AL | 30-60 | 23/09/2020 | lower part of active layer; mineral soil with dark organic inclusions |
| HUS_3 | 69° 0' 53.7978" | 133° 16' 45.951" | Turbel | | Pc | 60-90 | 23/09/2020 | samples from top 50cm of permafrost; stony till |

<u>Abbreviations:</u>

CRB_S: CRB Slump
EC: East Channel of Mackenzie River
HUS: Husky Slump
K: Klondike
P: Profile
T: Tuktoyaktuk
# (2 etc.): Number of soil profile

AL: active layer
ALc: active layer cryoturbated
PFc: permafrost cryoturbated
PFnc: permafrost non-cryoturbated

**Soil profiles and environmental conditions**

#2. Turbel-T-P1: tussock tundra on mineral lake silt

Coordinates: 69° 23' 22.5"N, 133° 27' 59.3"W

Location: drained-lake basin, 'Tuk 3' drained pingo lake site, 18 km WSW of Tuktoyaktuk

Date of sampling: 13 August 2019

Geomorphic context: old drained-lake basin elevated ~3–5 m above pingo drained-lake basin. Flattish terrain.

Vegetation: tussock tundra. Cottongrass, moss, lichen, sedges, Labrador tea, cloudberry, willow.

Organic soil: fibrous, ~20 cm thick.

Mineral soil: grey, silt-rich, strongly cryoturbated to depth of ~2 m (~1.5 m below top of permafrost). Horizontally laminated lake silts.

Active-layer thickness (ALT): 38 ± 1 cm (tussocky landsurface, probe, n=10); 47 ± 2 cm (ice-wedge top, vertical section, n=3)

Ground ice: abundant excess segregated ice and large ice wedges (≤1.5 maximum true width). Large size of ice wedges consistent with probable subaerial exposure of terrestrial surface since lake drainage (at least 1–2 ka?). Some pool ice bodies within wedge ice, indicating past local melting and refreezing. Lenticular to irregular-reticulate cryostructure in silt.

Sampling: vertical section of coastal bluff; samples 19-018-T-P1 to 19-040-T-P1.

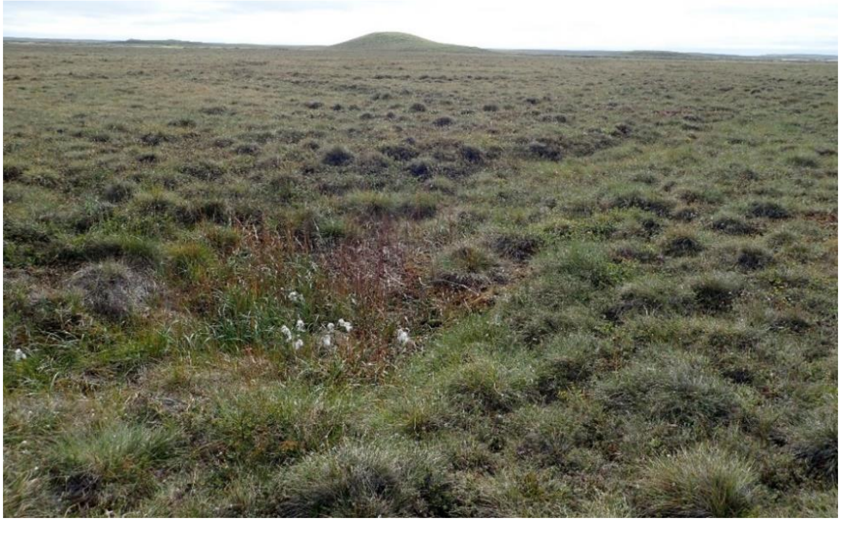

Supplementary Figure 4. **Tussock tundra near sampling site.** Cottongrass in ice-wedge trough. Pingo in distance.

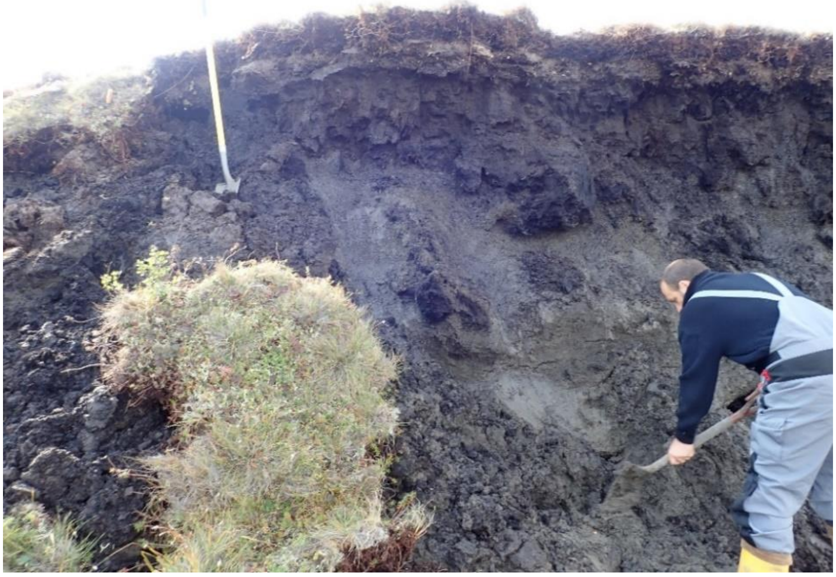

Supplementary Figure 5. **Vertical section through turbel in coastal bluff.** Black organic bodies in grey mineral soil indicate cryoturbation.

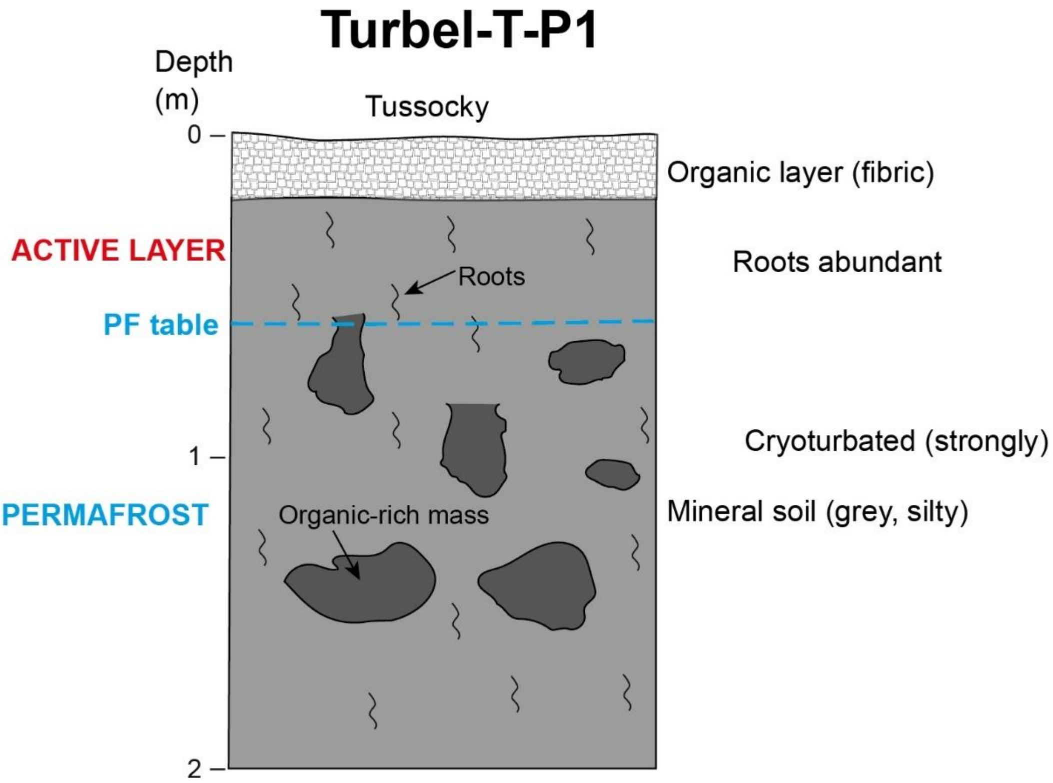


Supplementary Figure 6. **Schematic vertical section through Turbel-T-P1 exposed in coastal bluff.**

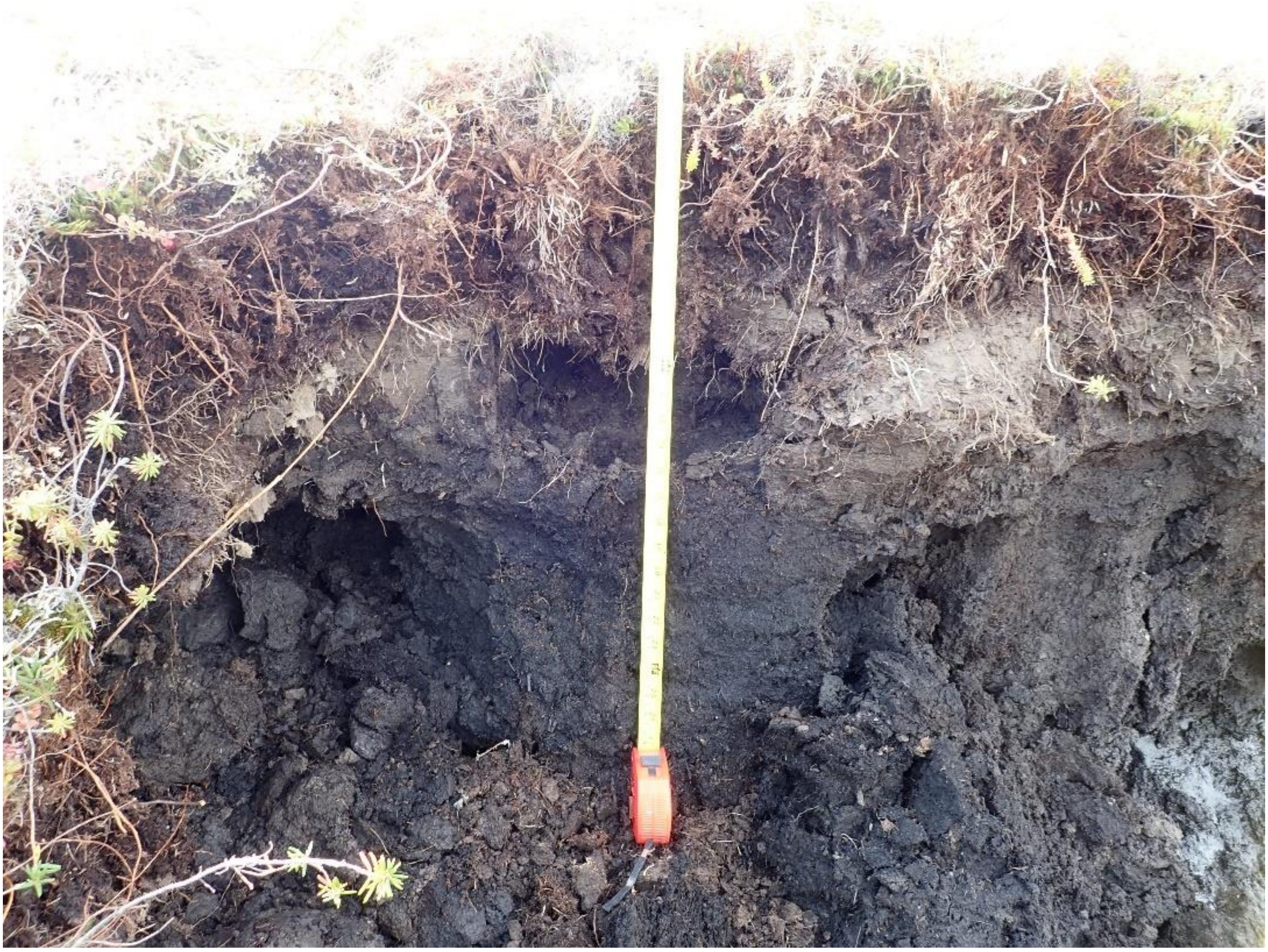

Supplementary Figure 7. **Vertical section through upper part of turbel.** Sample 19-018-T-P1 collected from hole behind centre of tape, 23–40 cm depth, beneath the brown organic layer.

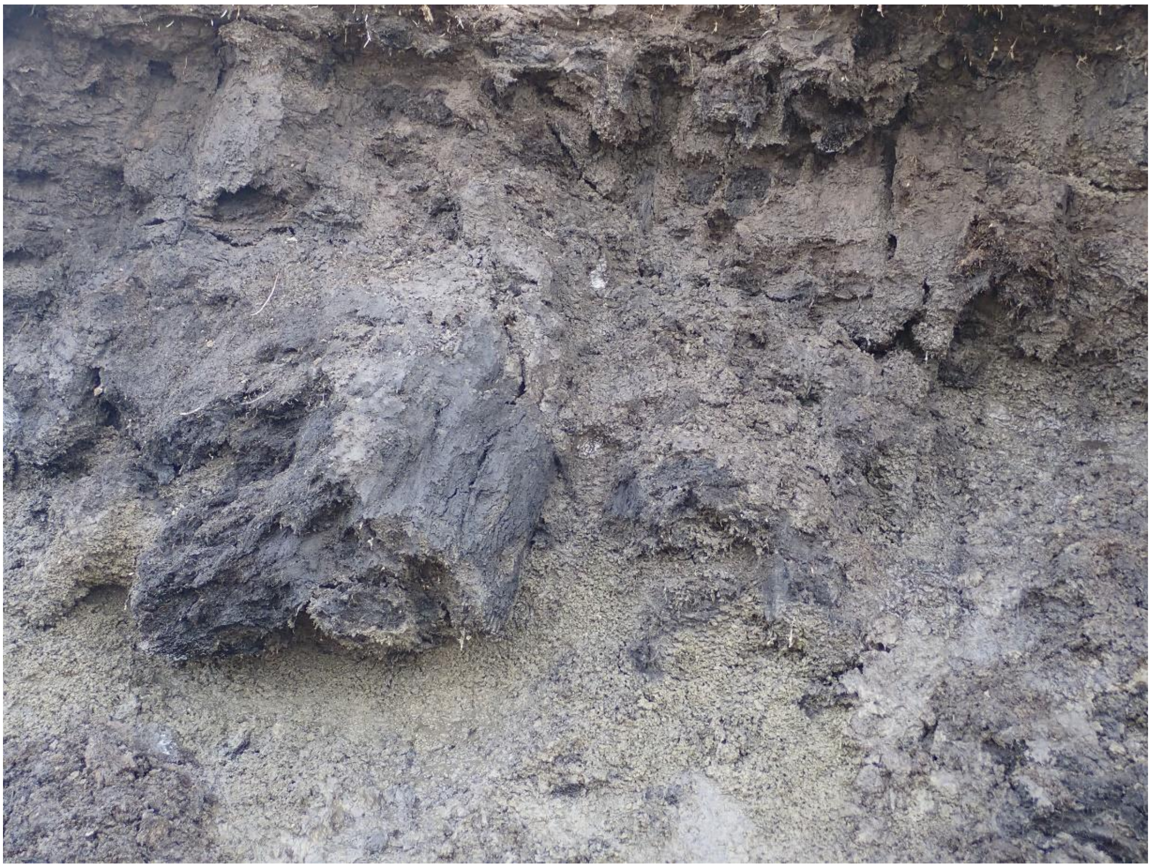

Supplementary Figure 8. **Cryoturbated near-surface permafrost.** Organic-rich bodies (black) in silty mineral soil (grey), ~1–2 m depth.

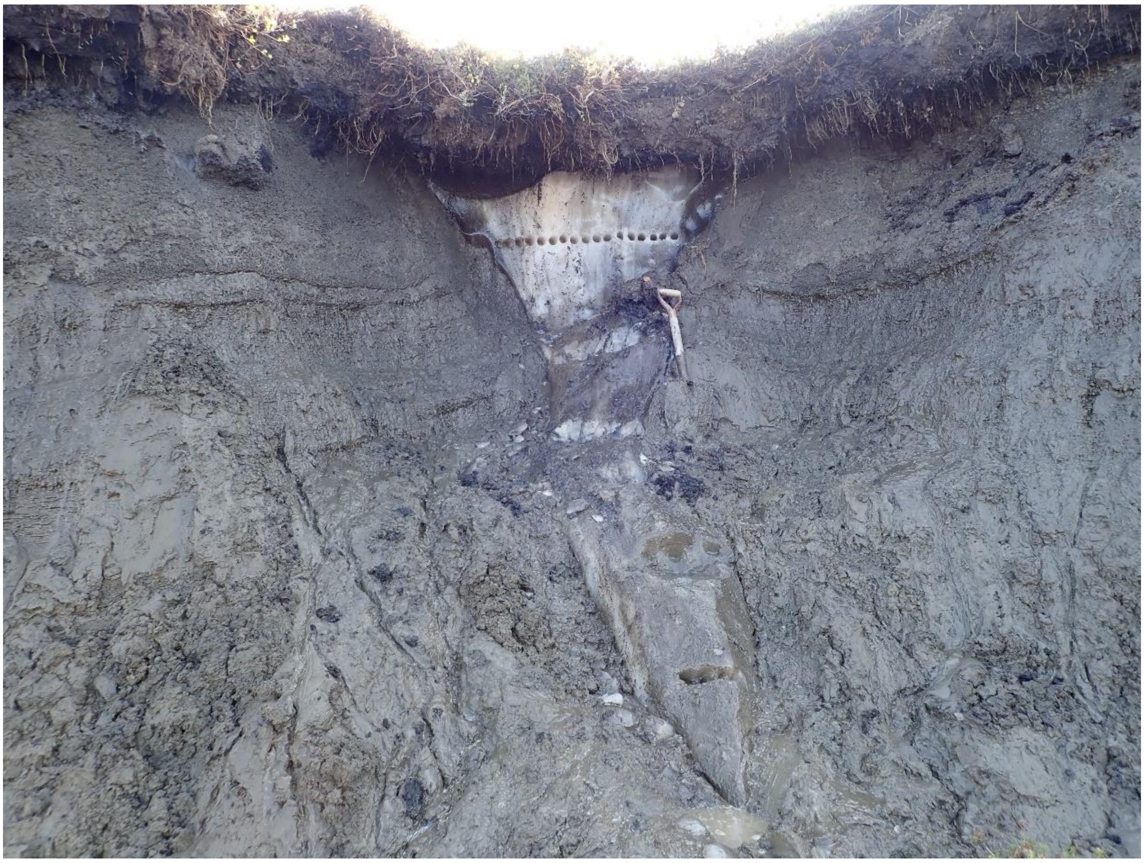

Supplementary Figure 9. **Ice wedge (white) within grey silty mineral soil (lacustrine).** Sample holes for stable isotopes (Thomas Opel). Spade for scale.

#3. Orthel-T-P2: erect-shrub tundra on organic lake silt

Coordinates: 69° 22' 53.6"N, 133° 17' 18.8"W

Location: 12 km southwest of Tuktoyaktuk

Date of sampling: 13 August 2019

Geomorphic context: drained-lake basin. High-centre ice-wedge polygons moderately developed.

Vegetation: erect-shrub tundra with cloudberry, Labrador tea, dwarf birch, cranberry, sedges.

Organic soil: black organic silt (lacustrine) at least 1.5 m thick.

Mineral soil: none observed.

ALT: 38 ± 2 cm (polygon centre, probe, n=10); 30 ± 4 cm (ice-wedge trough, probe, n=10)

Ground ice: wedge ice and pool ice are abundant in upper 2 m of section. No evidence of cryoturbation.

Sampling: vertical section of coastal bluff; samples 19-041-T-P1 to 19-057-T-P1

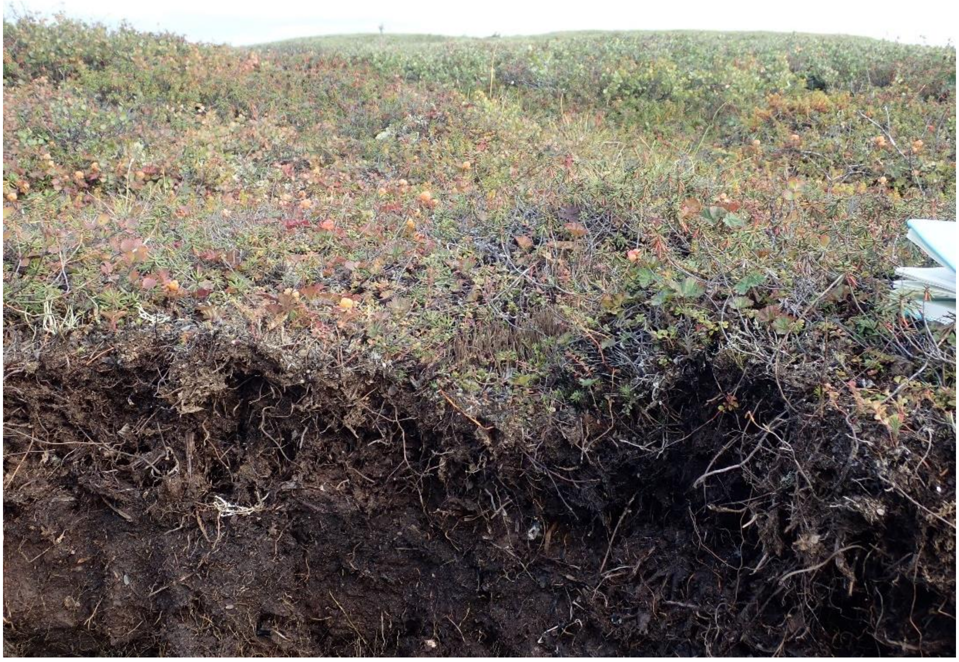

Supplementary Figure 10. **Shrub tundra at soil sampling site.** Cloudberry and Labrador tea abundant.

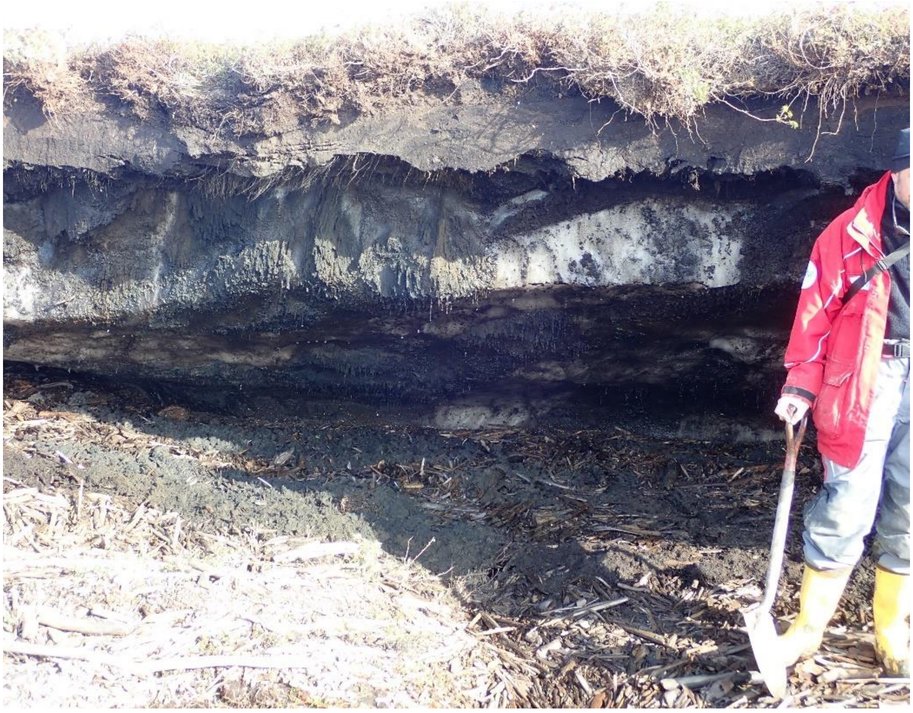

Supplementary Figure 11. **Vertical section through orthel in coastal bluff.** Wedge ice is white.

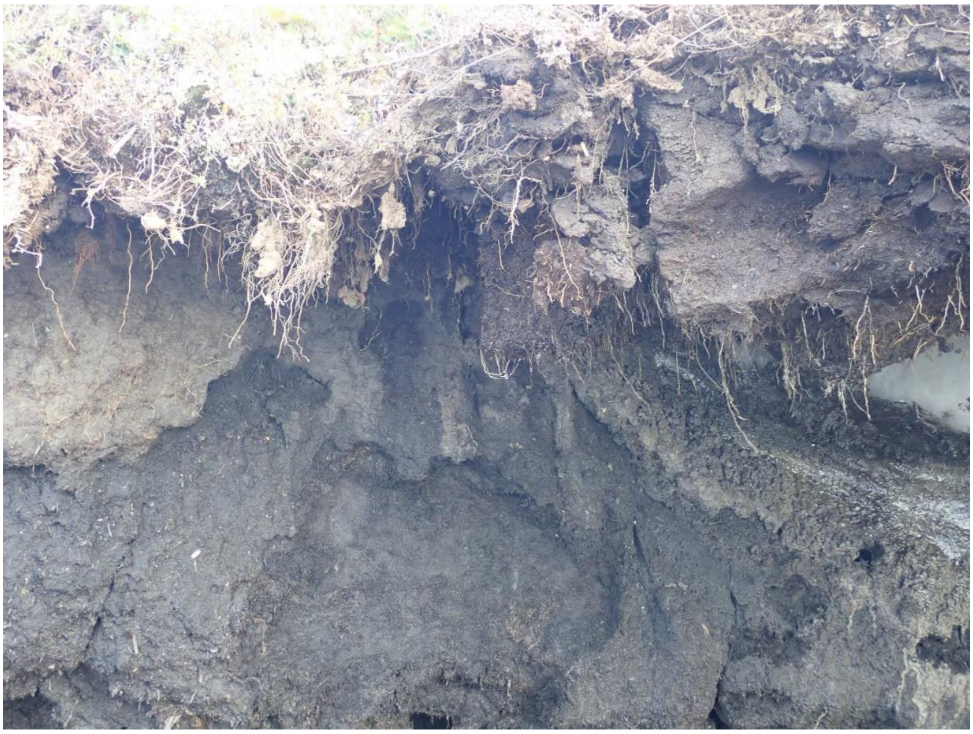

Supplementary Figure 12. **Vertical section through orthel.** Sample 19-041-T-P2 collected from 25–40 cm depth, and sample 19-042-T-P2 from 60–100 cm depth in black organic silt, approximately in centre of photograph. Section ~1.5 m high.

#4. Turbel-T-P3: erect-shrub tundra on till

Coordinates: 69° 25' 04.3"N, 133° 00' 36.4"W

Location: west side of Tuk Harbour, 1.2 km south of SE end of Tuk runway.

Date of sampling: 14 August 2019

Geomorphic context: undulating glacial moraine, covered by hummocks, 0.5–1 m diameter, ≤0.5 m high.

Vegetation: erect-shrub tundra comprising willow, alder, Labrador tea, crowberry, sedges, cloudberry, cranberry

Organic soil: dark brown to black, fibrous, 10 cm thick

Mineral soil: cryoturbated silt–clay diamicton (Toker Point till, colluvially reworked) above brown fine sand and silty sand (Kittgazuit Formation; aeolian)

ALT: 64 ± 5 cm (hummock top, probe, n=5); 39 ± 5 cm (organic-rich inter-hummock trough, probe, n=5)

Ground ice: not observed.

Sampling: vertical section of coastal bluff; samples 19-058-T-P1 to 19-073-T-P1

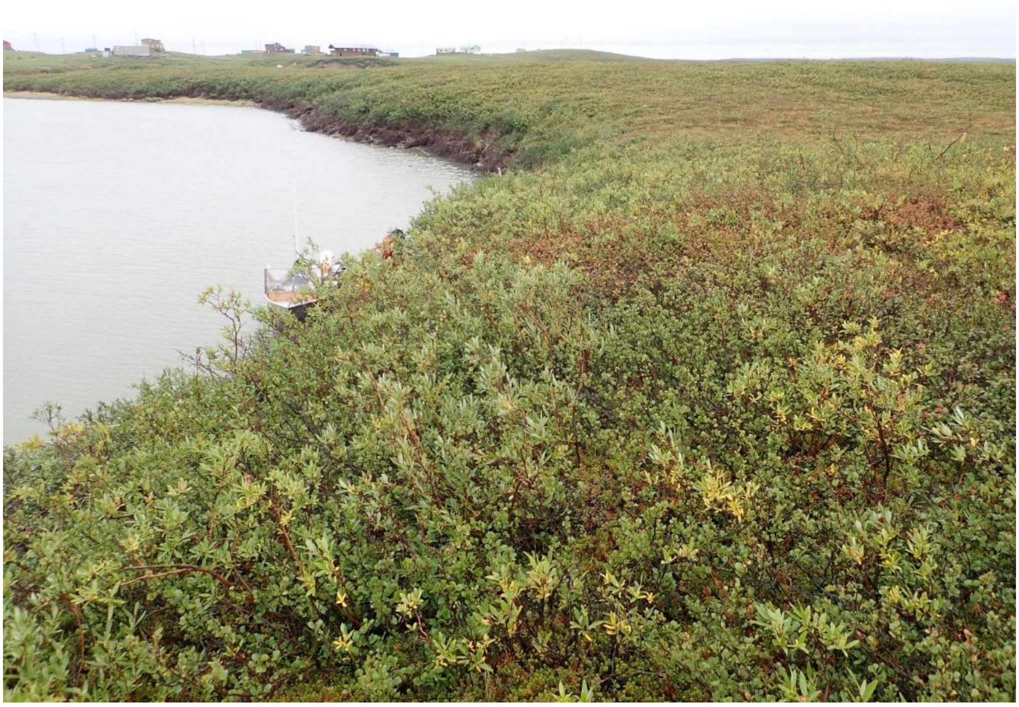

Supplementary Figure 13. **Shrub tundra near soil sampling site.** Willow abundant.

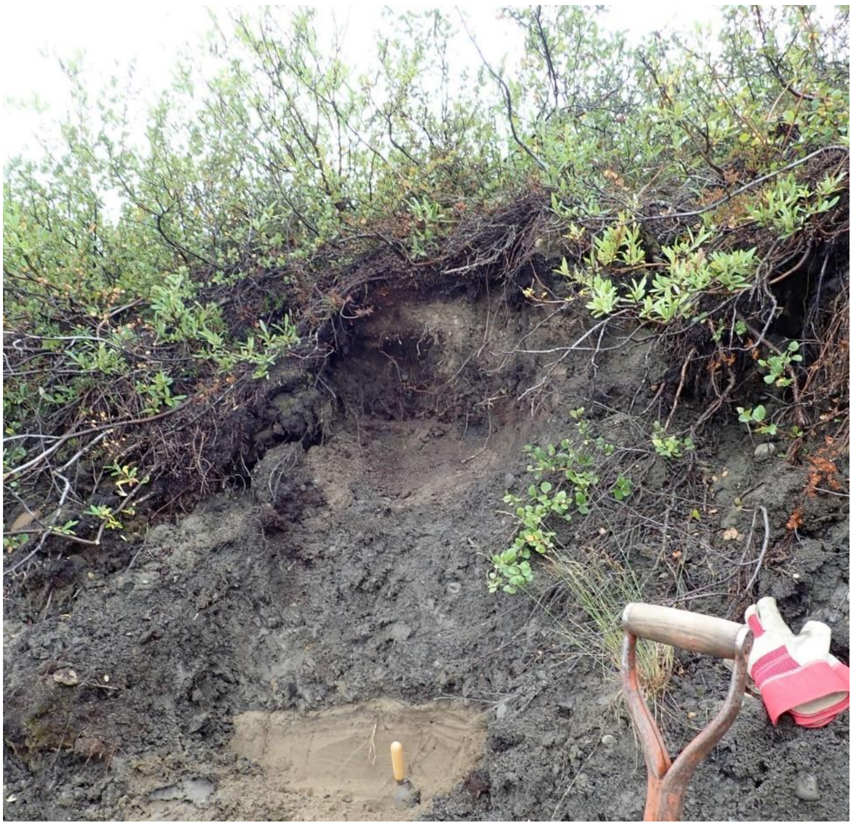

Supplementary Figure 14. **Vertical section through hummocky turbel in coastal bluff.**

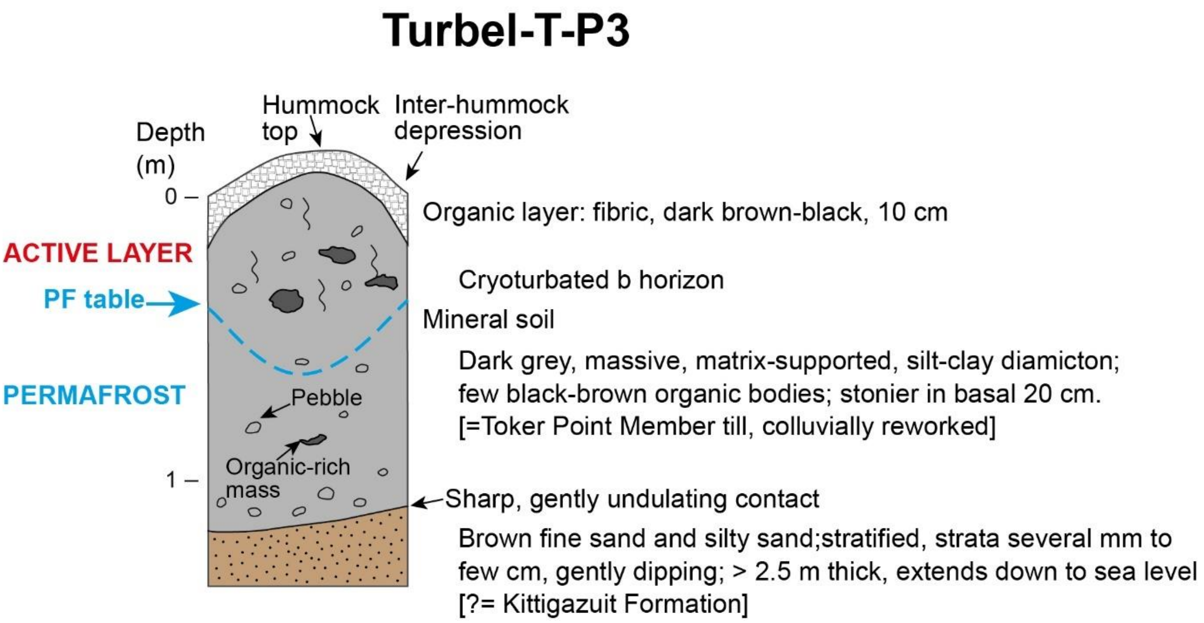


Supplementary Figure 15. **Schematic vertical section through Turbel-T-P3 exposed in coastal bluff.**

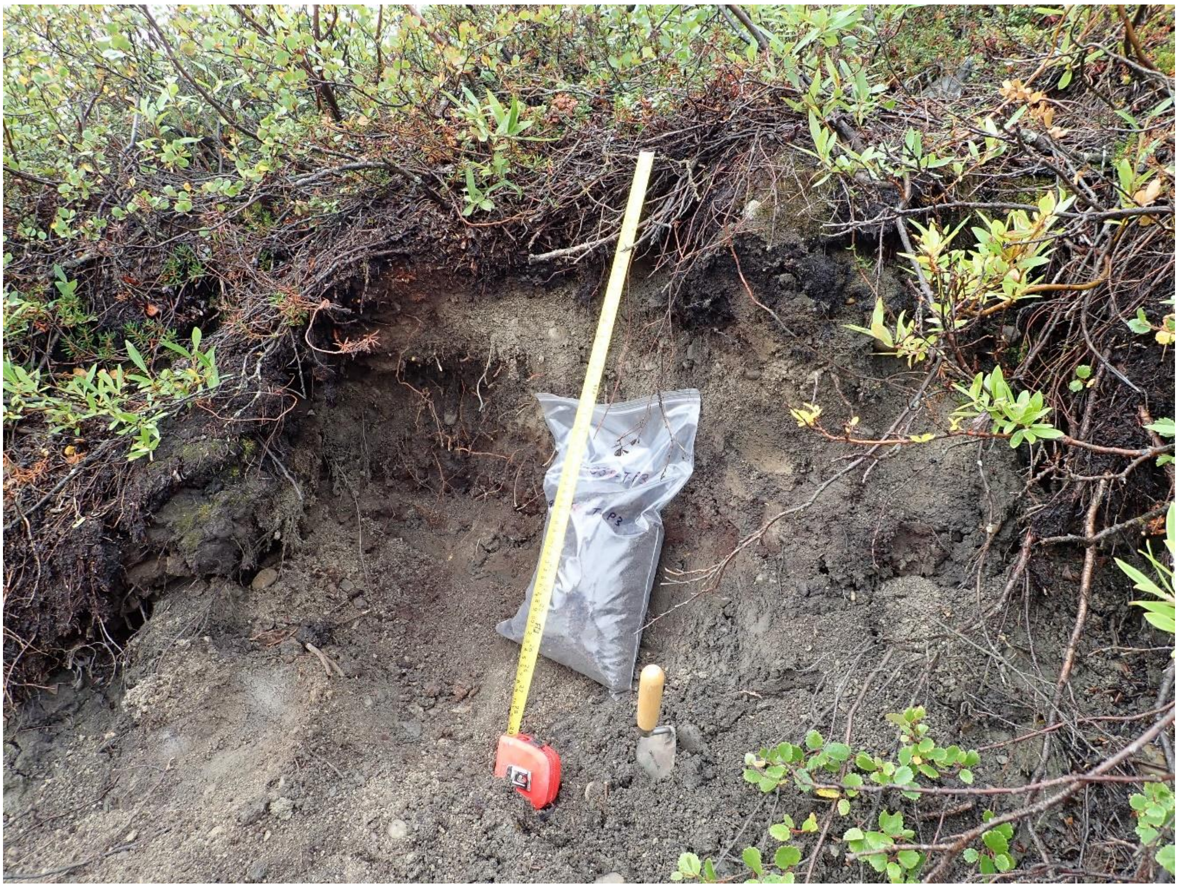

Supplementary Figure 16. **Vertical section through grey mineral soil (silt–clay diamicton) in turbel.** Sample 19-058-T-P3 collected at 20–45 cm depth.

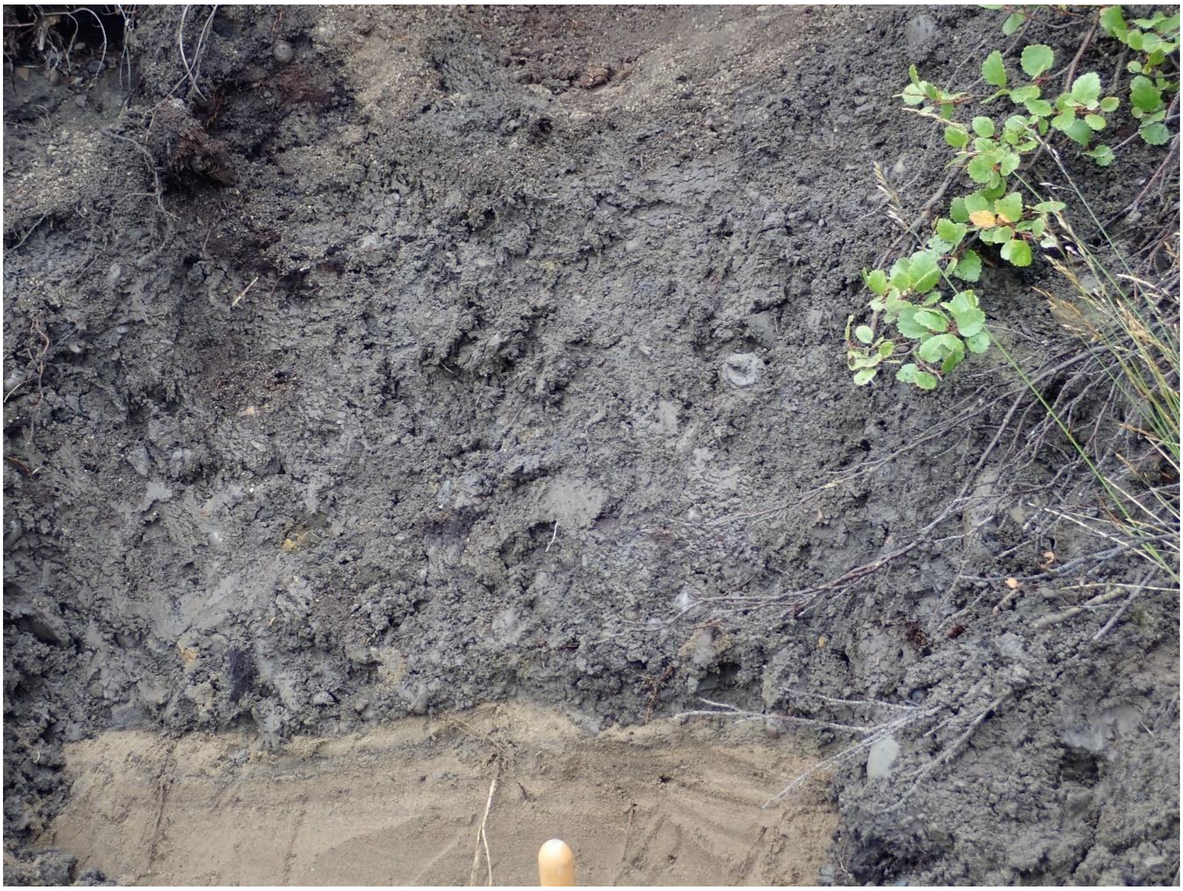

Supplementary Figure 17. **Vertical section showing grey silt–clay diamicton (till) above brown sand.** Sample 19-059-T-P3 collected at 90–110 cm depth, near base of diamicton.

#5. Turbel-T-P4: erect-shrub tundra on till

Coordinates: 69° 24' 28.7"N to 133° 00' 17.2"W

Location: west side of Tuk Harbour, 2.3 km south of SE end of Tuk runway.

Date of sampling: 14 August 2019

Geomorphic context: hummocky hillslope with gradient of few °. Undulating morainic topography as at previous site.

Vegetation: erect-shrub tundra comprising willow, Labrador tea, sedges, crowberry, alder, bearberry; moss in inter-hummock troughs.

Organic soil: 10 cm thick organic layer

Mineral soil: very dark grey to black, cryoturbated silty to peaty developed on silt–clay diamicton (till)

ALT: 51 ± 5 cm (hummock top, probe, n=5); 29 ± 5 cm (organic-rich inter-hummock trough, probe, n=5)

Ground ice: ice-rich in diamicton (till), but limited exposure prevented detailed observations.

Sampling: vertical section of coastal bluff; samples 19-074-T-P4 to 19-091-T-P4

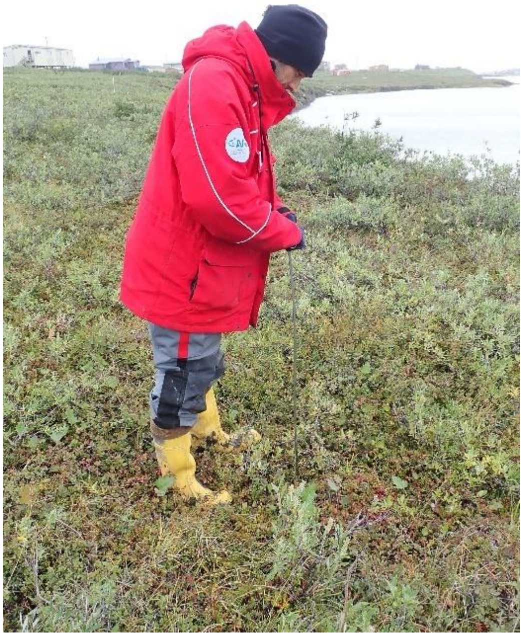

Supplementary Figure 18. **Shrub tundra near soil sampling site.** Active-layer probing.

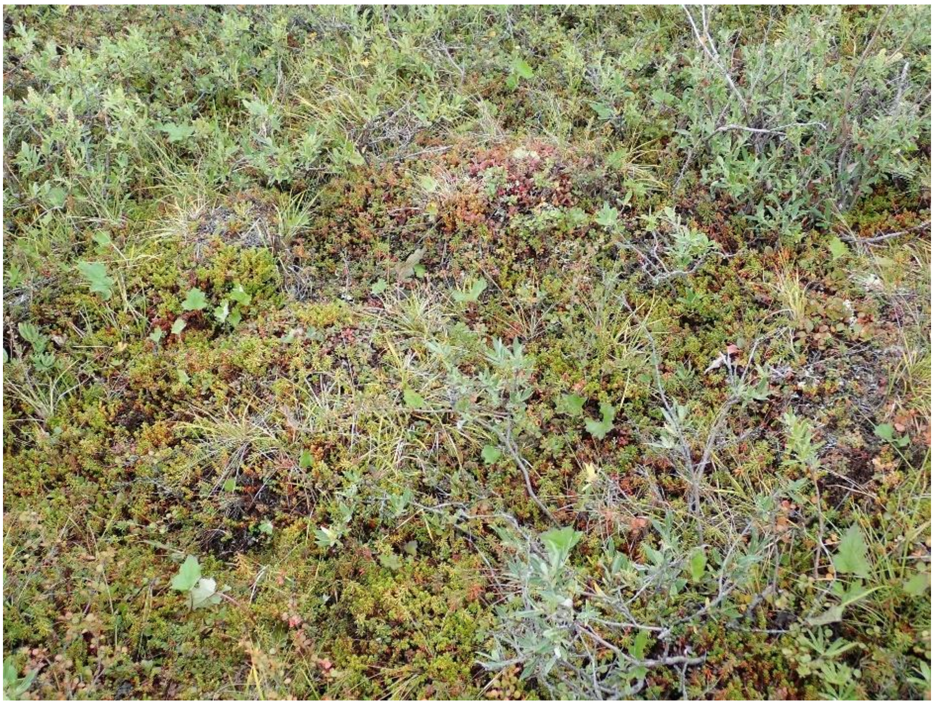

Supplementary Figure 19. **Hummocks on hillslope.**

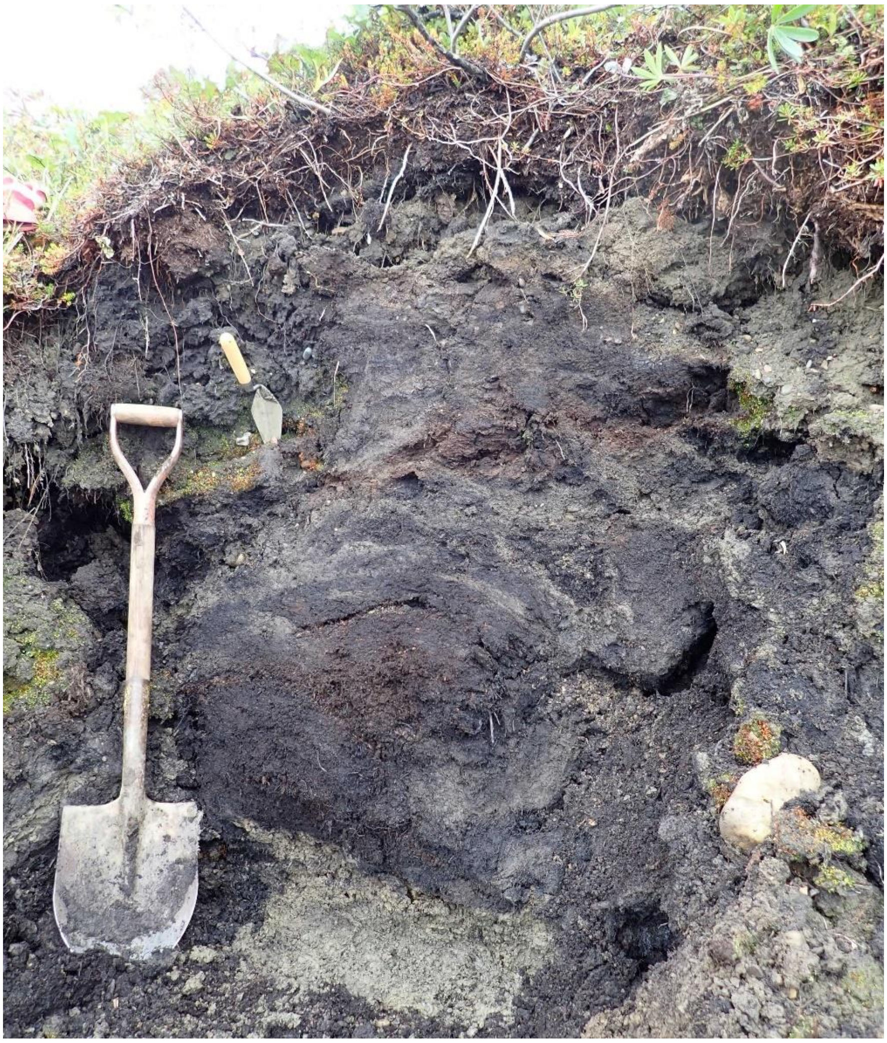

Supplementary Figure 20. **Vertical section through hummocky turbel in coastal bluff.** Spade for scale. Sample 19-074-T-P4 from organic-rich material at 0.3–0.45 m depth, sample 19-075-T-P4 from peaty material at 0.9–1.1 m depth, and sample 19-076-T-P4 from silt–clay diamicton (till) at 1.3–1.5 m depth (near base of spade).

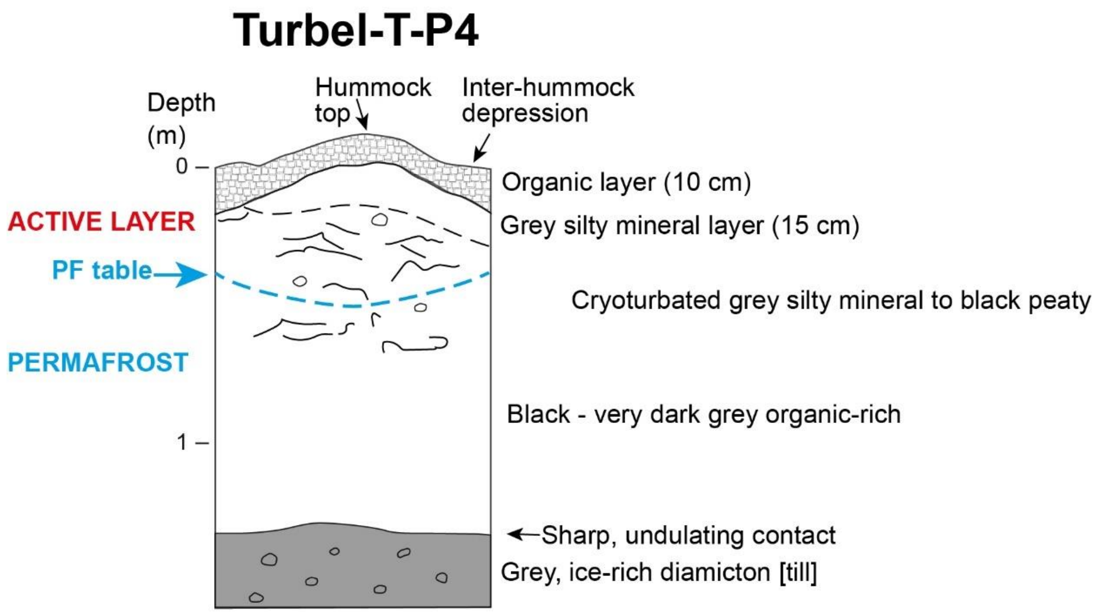


Supplementary Figure 21. **Schematic vertical section through Turbel-T-P4 exposed in coastal bluff.**

#6. Turbel-T-P5: erect-shrub tundra on till

Coordinates: 69° 24' 02.9"N, 132° 59' 46.0"W

Location: Reindeer Point, SW side of Tuk Harbour, 3.1 km SSE of SE end of Tuk runway.

Date of sampling: 15 August 2019

Geomorphic context: gently undulating moraine covered with hummocks ~1–2 m developed on Toker Point till. Numerous pebbles to boulders, coarsely crystalline, including granite, ?granodiorite, porphrytic gneiss = erratics probably derived from Canadian Shield.

Vegetation: erect-shrub tundra comprising willow, birch, cranberry, crowberry, bearberry, Lab. tea

Organic soil: as before.

Cryoturbated horizon: ~10 cm thick @ ~60 cm depth comprising discontinuous bodies of black peaty material, presumably concentrated at or near base of active layer by cryoturbation in hummocks, leading to burial of organic material as hummocks move downslope.

Mineral soil: silt–clay diamicton (Toker Point till) overlying grey sand, stratified (unknown stratigraphic affiliation).

ALT: 72 ± 9 cm (hummock top, probe, n=5); 48 ± 11 cm (organic-rich inter-hummock trough, probe, n=5)

Ground ice: not observed but limited time at site

Sampling: vertical section of coastal bluff; samples 19-092-T-P5 to 19-108-T-P5

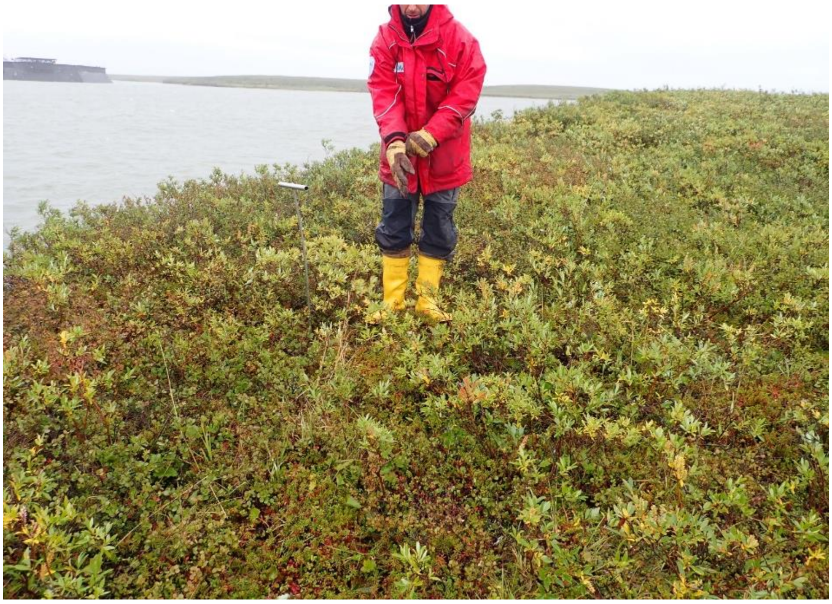

Supplementary Figure 22. **Shrub tundra near soil sampling site.** Active-layer probing.

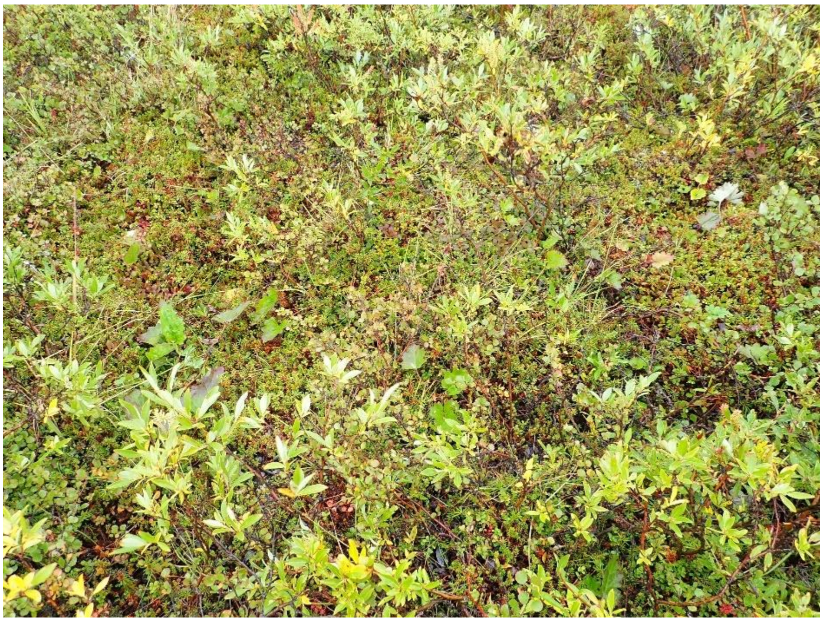

Supplementary Figure 23. **Vegetation on hummocks.**

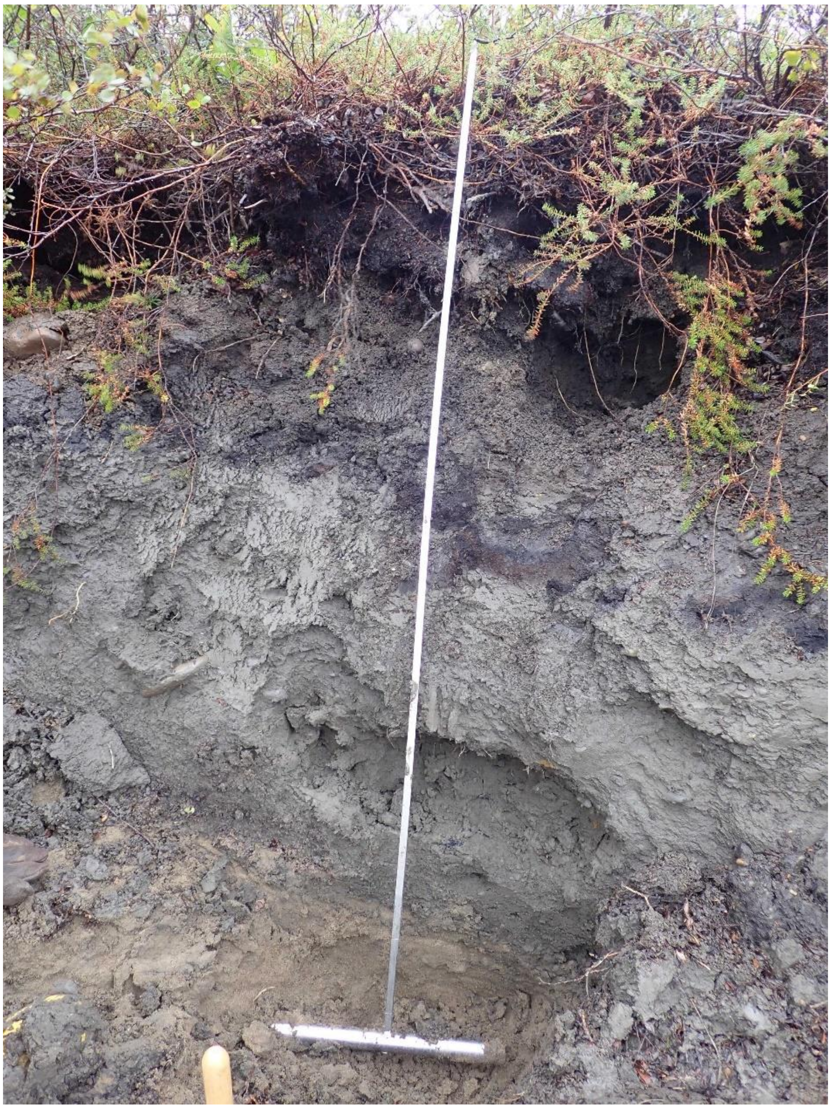

Supplementary Figure 24. **Vertical section through hummocky turbel in coastal bluff.** Note black cryoturbated horizon at ~ 60 cm depth, in lower part of active layer. 1.5-m-long active-layer probe for scale. Sample 19-092-T-P5 from silt–clay & organic bodies at 0.3–0.5 m depth (upper hole to right of probe), and sample 19-093-T-P5 from grey silt–clay diamicton (till) at 0.95–1.1 m depth (hole behind lower part of probe). Insufficient organic material to sample from near-surface permafrost. Sand (brown) exposed at base of section.

<u>#7. Histel-T-P6: erect-shrub tundra on organic lake silt</u>

Coordinates: 69° 24' 05.6"N, 133° 02' 46.3"W

Location: 3.3 km southwest of SE end of Tuktoyaktuk runway

Date of sampling: 16 August 2019

Geomorphic context: high-centre ice-wedge polygons with relief ≤1 m; standing water in some trough. Sea inundated former lake beside site. Interpreted as old drained-lake basin truncated by younger lake now inundated by sea.

Vegetation: erect-shrub tundra: dwarf birch, lichen, cloudberry, Labrador tea, cranberry, crowberry on polygons; sedges and mosses in troughs

Organic soil: black organic layer 15 cm thick above brown fibric peat 15–30 cm depth (wetland) above black humic organic silt to at least 3 m depth (lacustrine)

Mineral soil: none observed

ALT: 39 ± 3 cm (centre of high-centre polygons, probe, n=10); 26 ± 4 cm (troughs between high-centre polygons, probe, n=10)

Ground ice: none observed

Sampling: vertical section of coastal bluff; samples 19-109-T-P6 to 19-125-T-P6

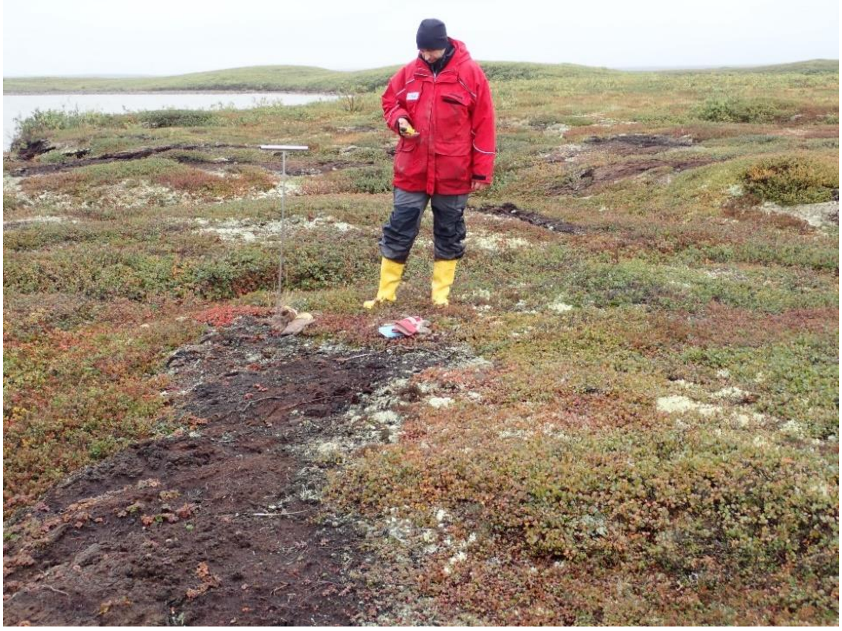

Supplementary Figure 25. **Shrub tundra on high-centre polygons near soil sampling site.** Active-layer probing.

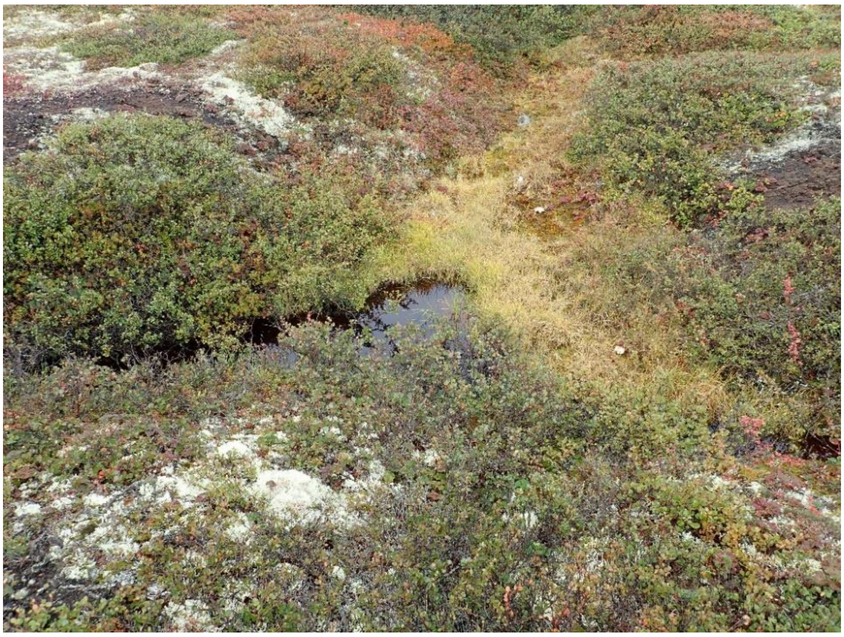

Supplementary Figure 26. **Sedgy pond in trough between high-centre polygons near soil sampling site.**

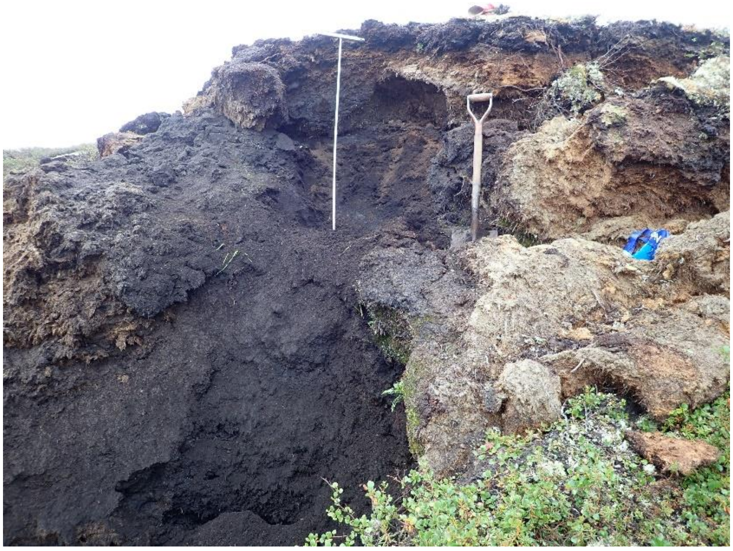

Supplementary Figure 27. **Vertical section through histel in coastal bluff.** 1.5-m-high active-layer probe for scale.

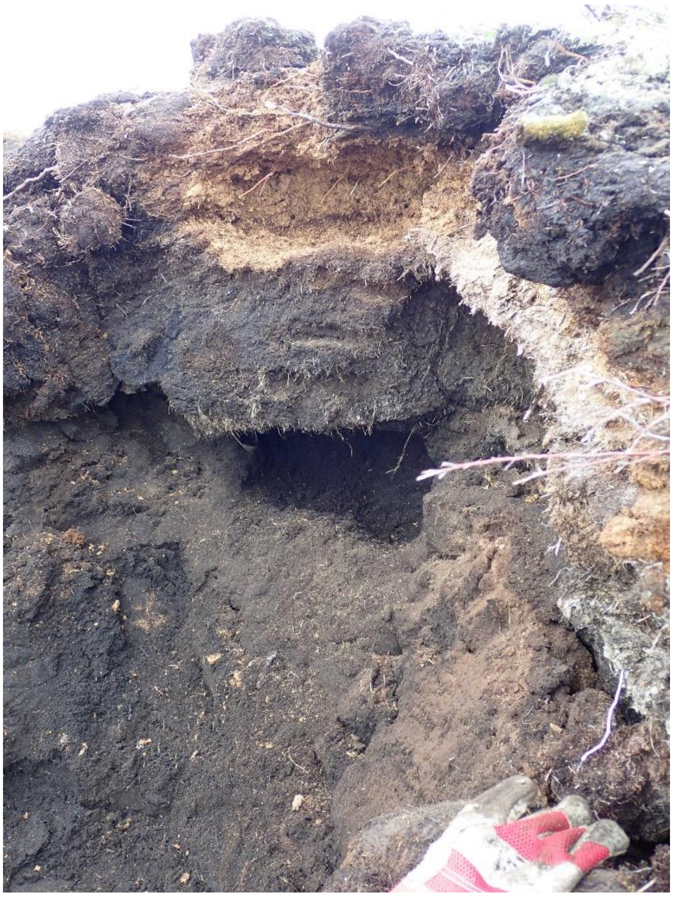

Supplementary Figure 28. **Sampling positions in histel.** Sample 19-109-T-P6 (brown fibrous wetland peat) collected at 0.15–0.30 m depth (upper hole), sample 19-110-T-P6 (black organic lake silt) from 0.7–0.9 m depth (lower hole).

## Palaeoenvironmental reconstruction of sites around Tuktoyaktuk Harbour:

Time (T)1: deposition of grey sand, probably waterlain, pre-glacial, but uncertain about exact process or age

T2: Toker Point Stade glaciation ~17–15 ka ⇨ deposition of veneer of Toker Point till above grey sand

T3: lake developed ⇨ deposition of black organic silt (black)

T4: drainage of lake ⇨ wetland ⇨ accumulation of mossy peat (brown)

T5: ice-wedge development, initially probably as low-centre polygons ⇨ moss peat

T6: partial melt of ice wedges ⇨ high-centre polygons ⇨ drying of tops of polygons ⇨ organic layer

#8. Orthel-EC-P2: old burn spruce forest on alluvium

Coordinates: 68° 26' 11.9"N, 133° 49' 03.3"W

Location: left bank of East Channel, Mackenzie River, ~10 km NNW of Inuvik

Date of sampling: 19 August 2019

Geomorphic context: flattish terrain ~4 m above river level.

Vegetation: open-canopy white spruce, alder, willow, blueberry, moss, bearberry, crowberry, horsetail. Alder and willow shrubs 1–2 m high dominant between spruce trees. Cut spruce stumps common (firewood?). Hummocks probably related to vegetation growth and old logs.

Organic soil: Well developed soil horizons: little evidence of cryoturbation, just minor folding. Burn layer 6–8 cm depth, black, includes charcoal. Fire did not burn intensely because some O horizon is preserved beneath burn layer. Probably a ground fire rather than a very hot crown fire.

Mineral soil: grey sand and silty sand, horizontally to subhorizontally stratified; strata parallel to wavy, 1 mm to several mm thick (alluvium); abundant roots penetrating down from soil.

ALT: 56 ± 6 cm (probe, n=10)

Ground ice: none observed

Sampling: vertical section of riverbank. Samples 19-126-EC-P2 to 19-142-EC-P2

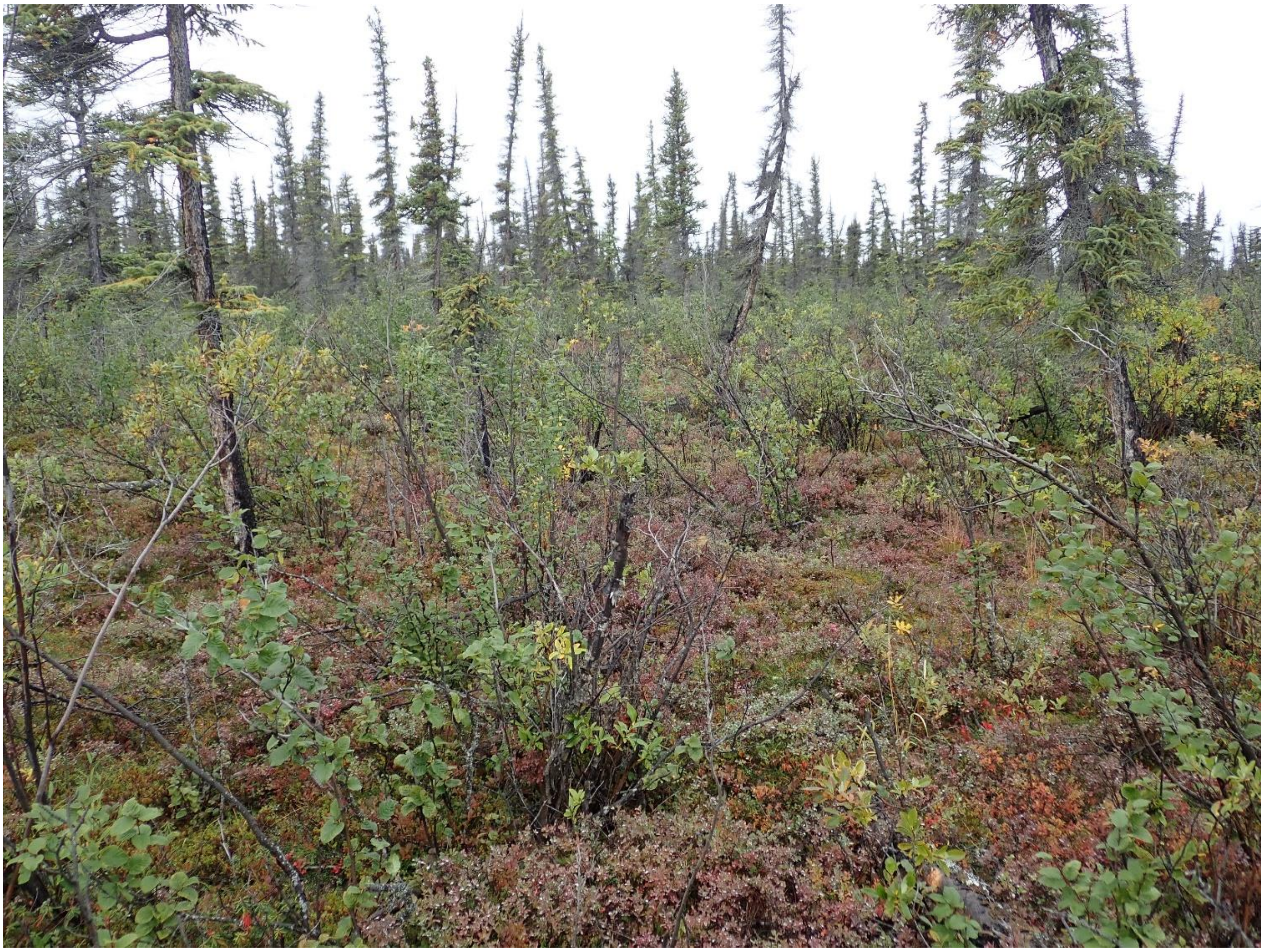

Supplementary Figure 29. **Old, open spruce forest near sampling site.**

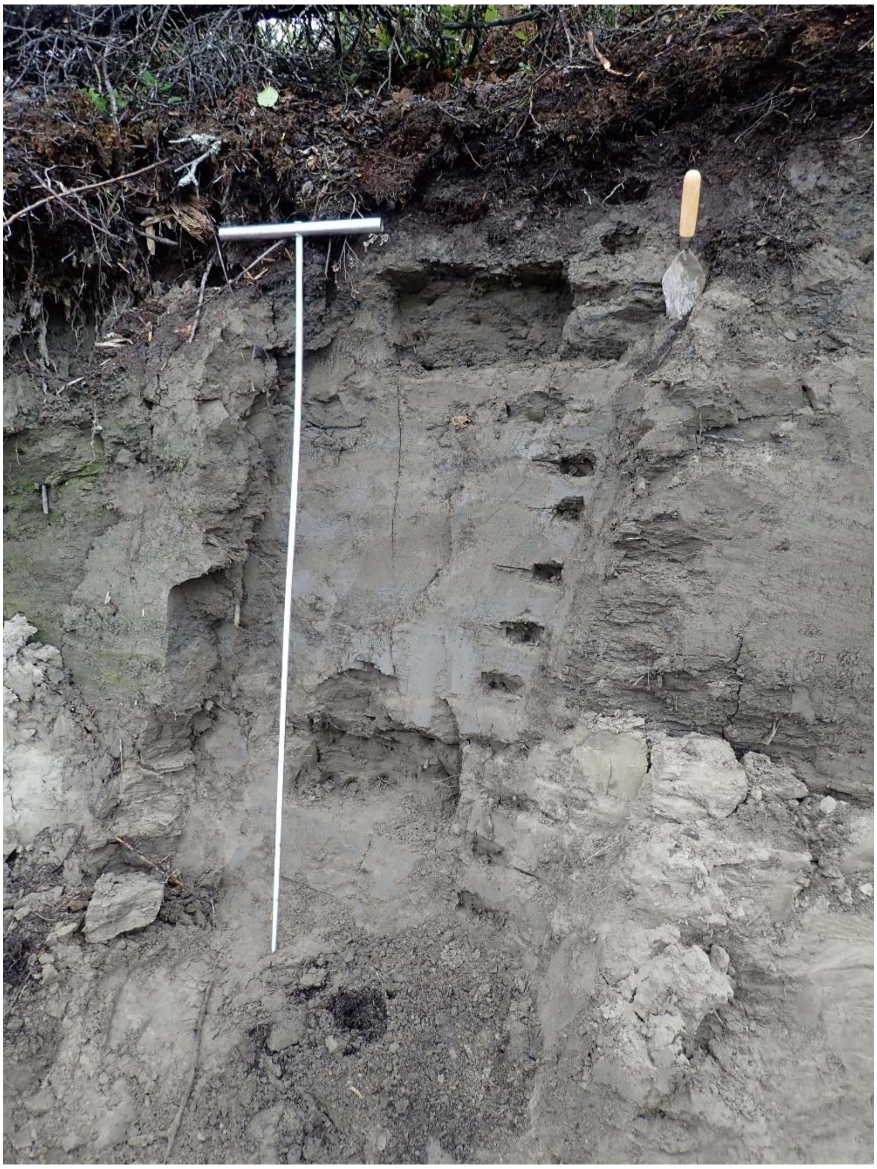

Supplementary Figure 30. **Vertical section through orthel.** Large soil samples 19-126-EC-P2 from 0.35–0.55 m depth from Bg–Bgw horizon, silty (large hole near top of active-layer probe) and 19-127-EC-P2 from 1.2–1.4 m depth from C horizon (large hole near bottom of probe). Small holes mark position of soil samples collected at 10-cm vertical intervals.

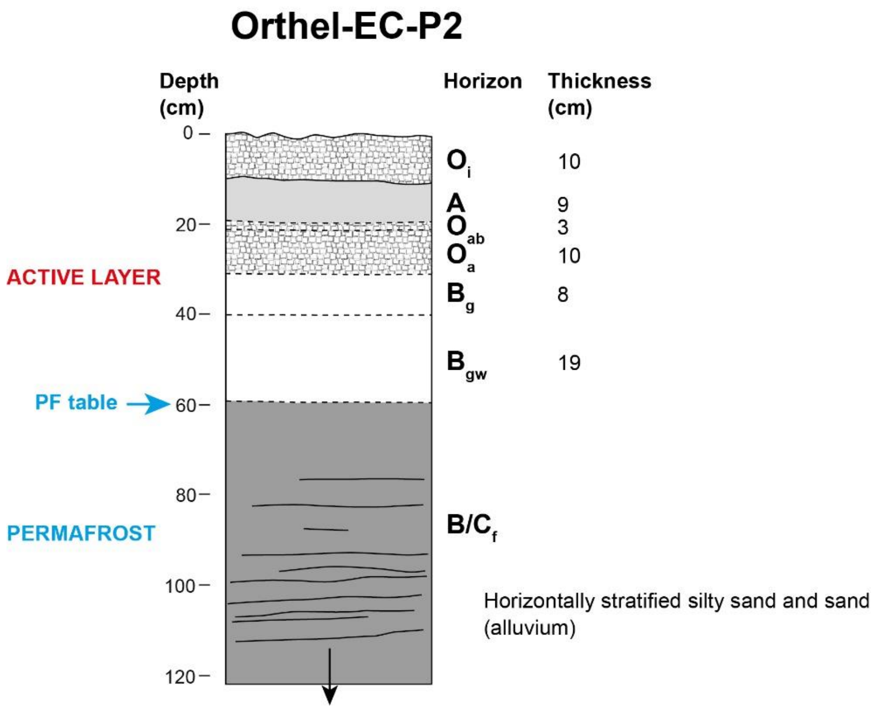


Supplementary Figure 31. **Schematic vertical section through orthel exposed in riverbank.**

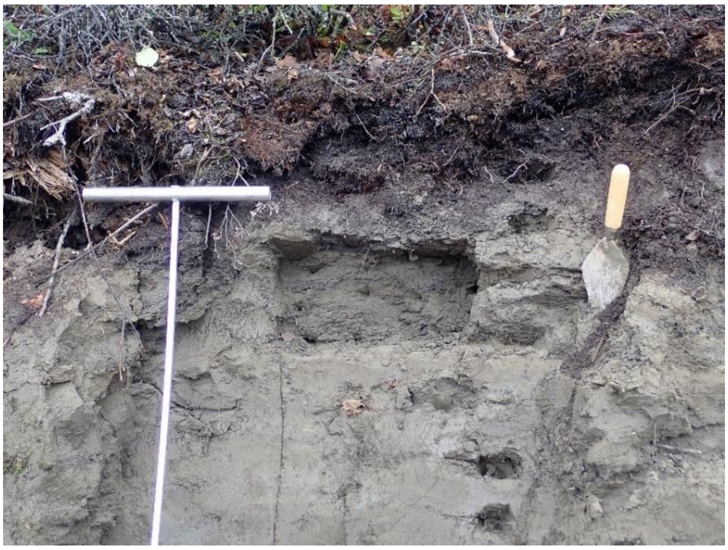

Supplementary Figure 32. **Close-up of upper ~80 cm of orthel.** Sample 19-126-EC-P2 collected at 0.35–0.55 m depth (large hole). Small holes are spaced at 10-cm intervals, from which samples collected.

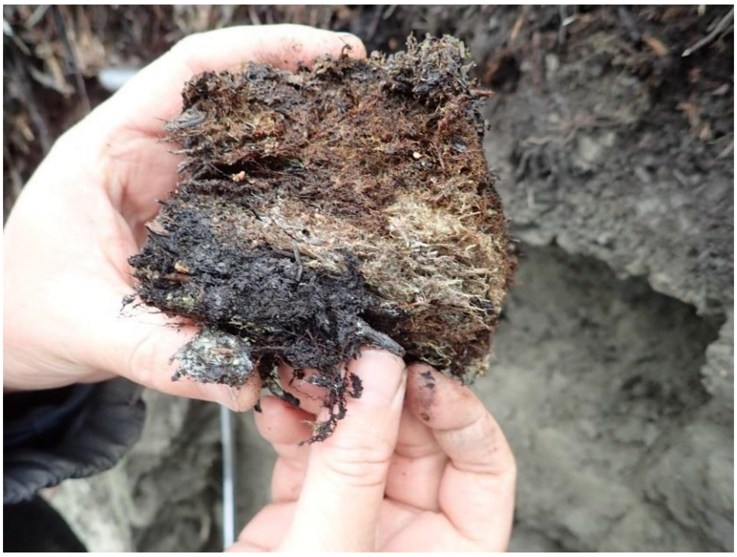

Supplementary Figure 33. **Close-up of buried burn layer (black).**

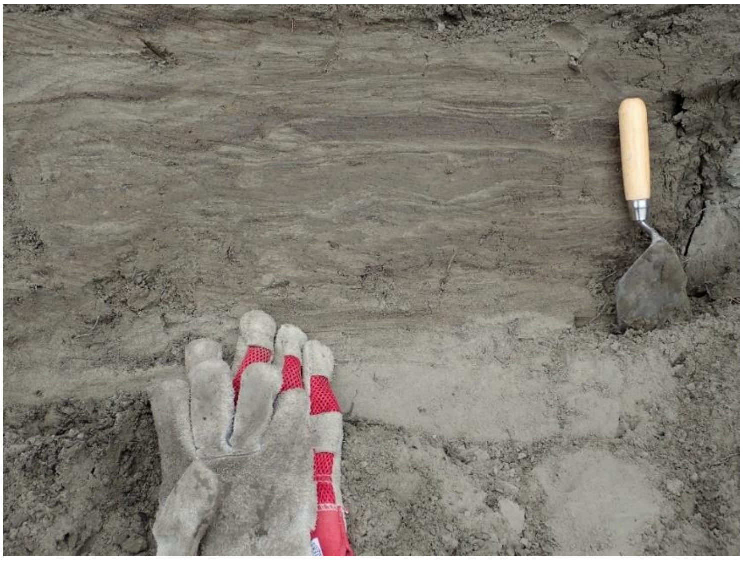

Supplementary Figure 34. **Wavy laminae (current ripples) in sand and silty sand (alluvium) of mineral soil.**

#9. Orthel-EC-P3: old spruce–lichen forest on alluvium

Coordinates: 68° 28' 18.3"N, 133° 51' 09.4"W

Location: left bank of East channel, Mackenzie River, ~14 km NNW of Inuvik

Date of sampling: 19 August 2019

Geomorphic context: landsurface ~4.5 m above river level

Vegetation: old white spruce forest, open canopy; willow, Lab. tea, alder, blueberry, lichen (*Cladonia* sp.), horsetail, cranberry, crowberry. Older forest than P2 (previous site) because abundant lichen. ⇨ Climax vegetation.

Organic soil: buried soil horizon 25–26 cm overlain by A horizon; burial probably due to flooding

Mineral soil: sand and silty sand to sandy silt, horizontally to subhorizontally stratified as at previous site P2. Abundant detrital wood fragments with rounded ends, presumably transported by river and deposited with alluvium.

ALT: 57 ± 5 cm (probing by Hugelius, n=10), 54 ± 11 cm (probing by Opel, n=10), 51 ± 10 cm (probing by Wagner, n=10), 60 ± 12 cm (probing by Murton, n=10), 59 ± 9 cm (probing by boat operator, n=10)

Ground ice: not observed, though loose, crumbly layer at ~60–80 cm depth, ?due to melt of ice at top of permafrost.

Sampling: vertical section of riverbank. Samples 19-143-EC-P3 to 19-159-EC-P3

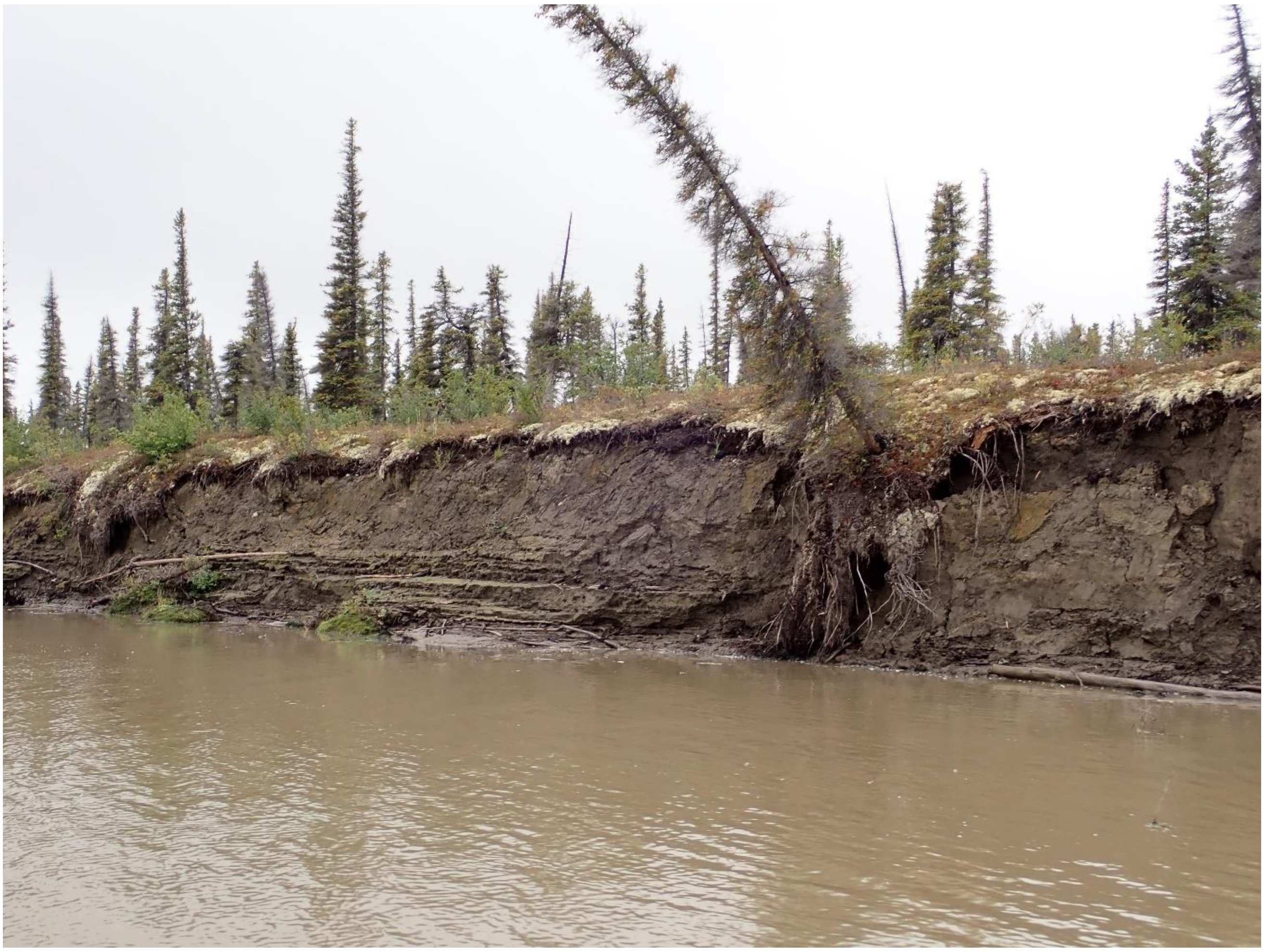

Supplementary Figure 35. **Old, open spruce forest near sampling site.** Tilted ('drunken') spruce tree on bank. Abundant lichen (light-coloured patches) on land surface.

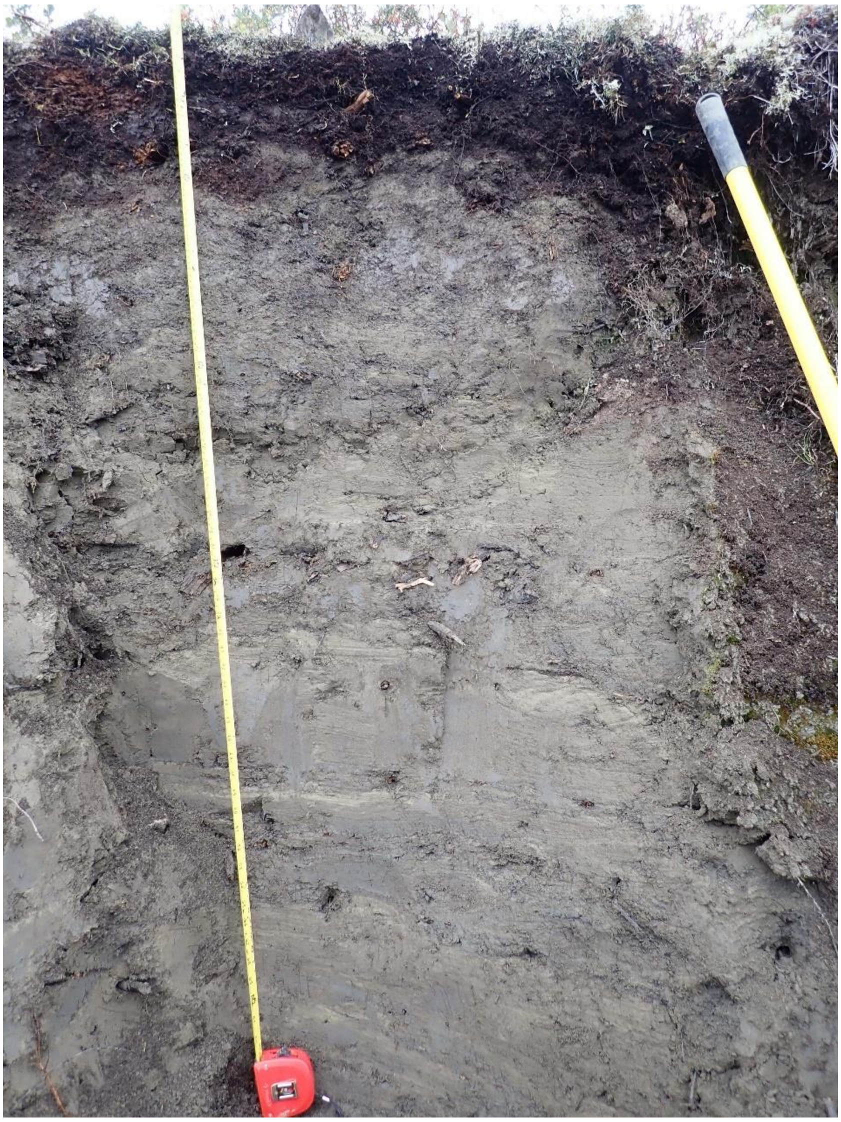

Supplementary Figure 36. **Vertical section ~1.5 m deep through orthel.** Large soil samples 19-143-EC-P3 at 0.30–0.50 m depth and 19-144-EC-P3 at 0.90–1.10 m depth.

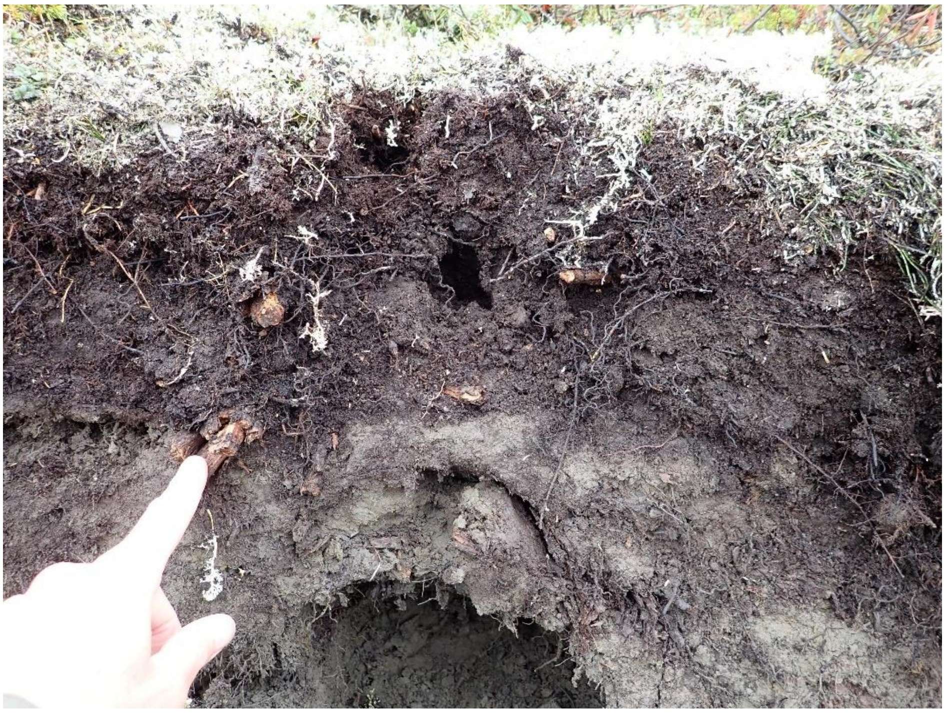

Supplementary Figure 37. **Close-up of organic layer and upper part of orthel.** Large soil sample 19-143-EC-P3 from silty Bgs horizon at 0.3–0.50 m depth (large hole in bottom centre).

#10. Orthel-EC-P4: old spruce forest on alluvium

Coordinates: 68° 31' 17.0"N, 133° 51' 29.0"W

Location: right bank of East Channel, Mackenzie River, ~19 km NNW of Inuvik

Date of sampling: 20 August 2019

Geomorphic context: flattish landsurface ~4 m above river level

Vegetation: open white spruce forest with willow and alder shrubs, and blueberry, sedges, bearberry, horsetail, moss, Lab. tea, limited lichen, crowberry. Vegetation hummocks.

Organic soil: O horizon (10 cm) above A horizon (15 cm). No sign of buried O horizons.

Mineral soil: dark grey, massive, silt–clay. Mottles (~1 cm) ~20–40 cm depth. Very limited evidence for cryoturbation: few dark grey bodies ~30 cm depth, but horizontal to gently undulating lower contact of A horizon indicates minimal cryoturbation. Wood fragments ≤few cm diameter, ≤few tens cm long, horizontal to gently dipping, rounded edges (detrital wood in alluvium). Well-developed stratification below depth of ~1.2 m: horizontal to subhorizontal, gently undulating, few mm to several cm thick, laterally continuous for commonly few to several tens cm (therefore not disrupted by cryoturbation).

ALT: 92 cm to frost table in soil pit; no active-layer probe available

Ground ice: none observed, but standing water in bottom of soil pit (due to seepage and/or melt of ground ice). In vertical section: loose, friable layer at ~50–70 cm depth with platy structure comprising angular, tabular peds separated by horizontal fissures spaced few to several mm vertically apart (former lenticular cryostructure).

Sampling: soil pit and vertical section of riverbank. Samples 19-160-EC-P4 to 19-176-EC-P4

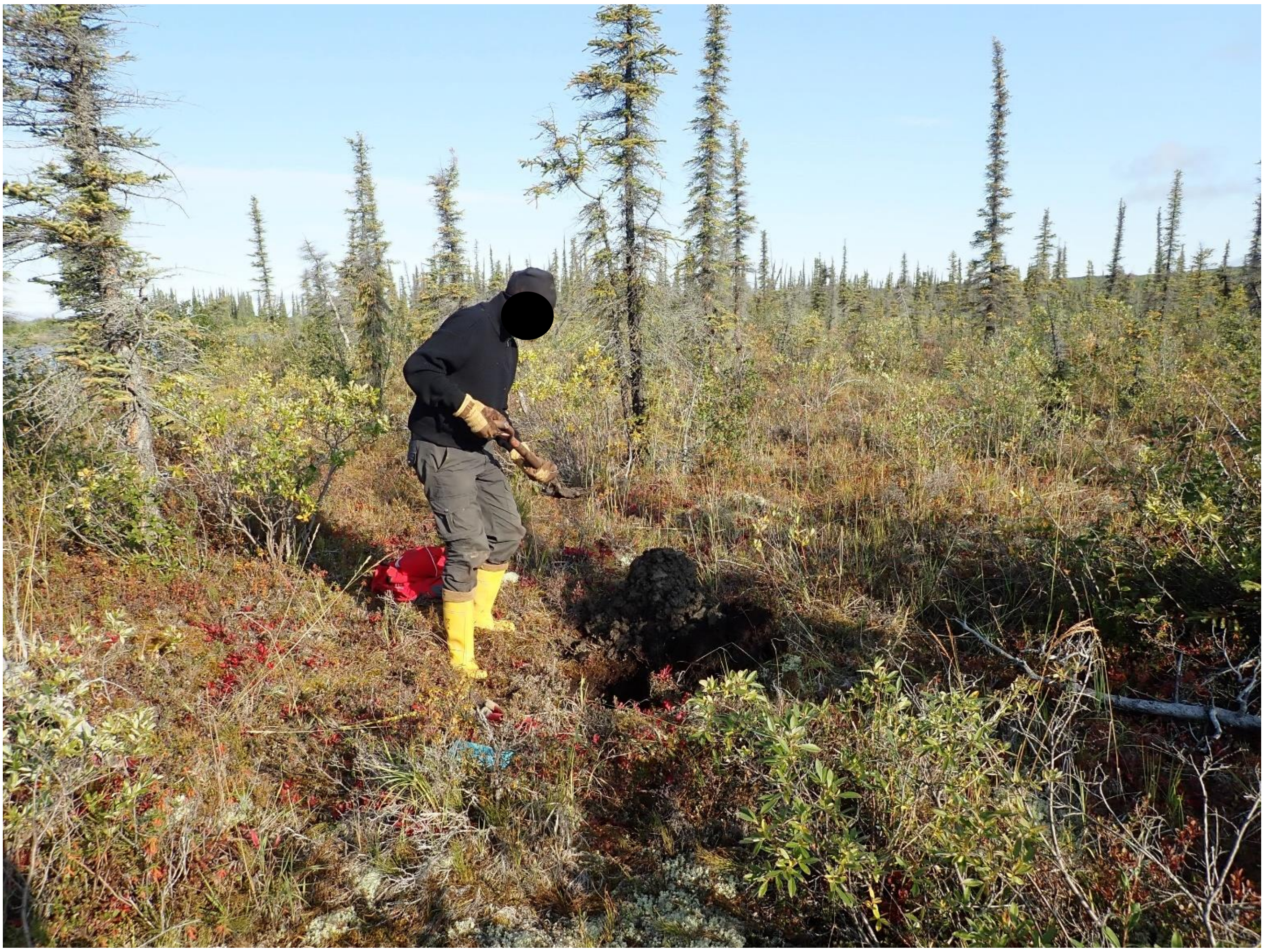

Supplementary Figure 38. **Old, open spruce forest near sampling site.** Soil pit in centre.

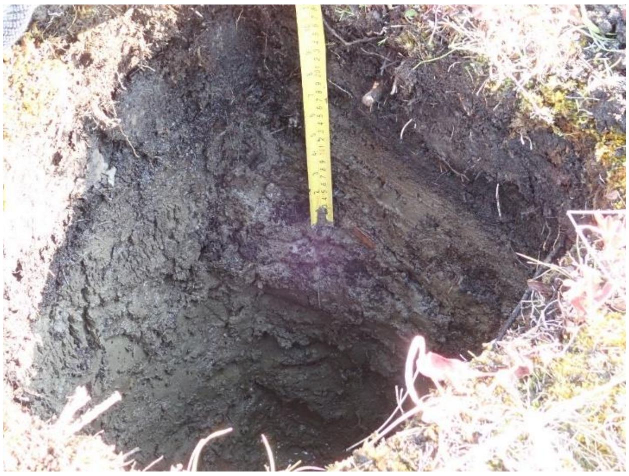

Supplementary Figure 39. **Orthel in soil pit 92 cm deep.** Wood fragment (brown) at 30 cm depth.

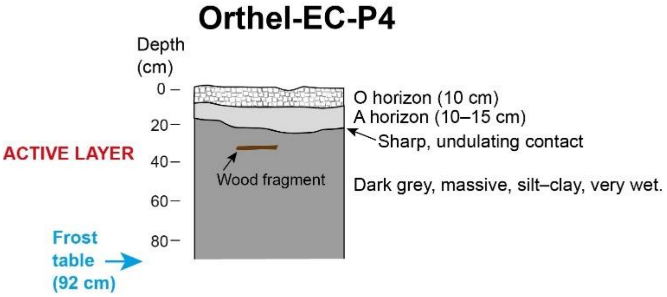


Supplementary Figure 40. **Schematic vertical section through of orthel exposed in soil pit.**

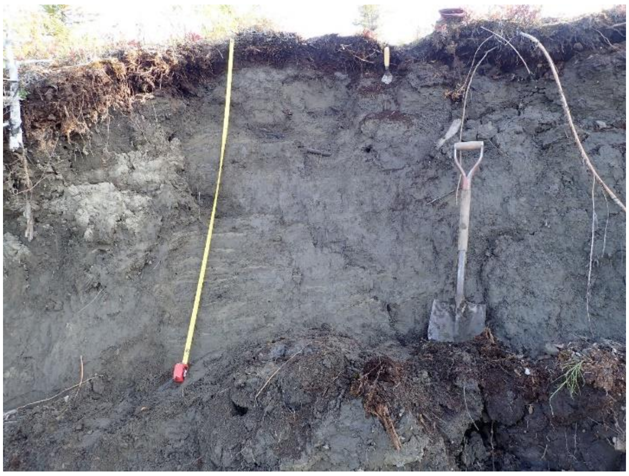

Supplementary Figure 41. **Vertical section through orthel.** Large soil samples 19-160-EC-P4 from Bg horizon at 0.3–0.5 m depth and 19-161-EC-P4 from C horizon, sandy silt (alluvium) at 1.1–1.3 m depth.

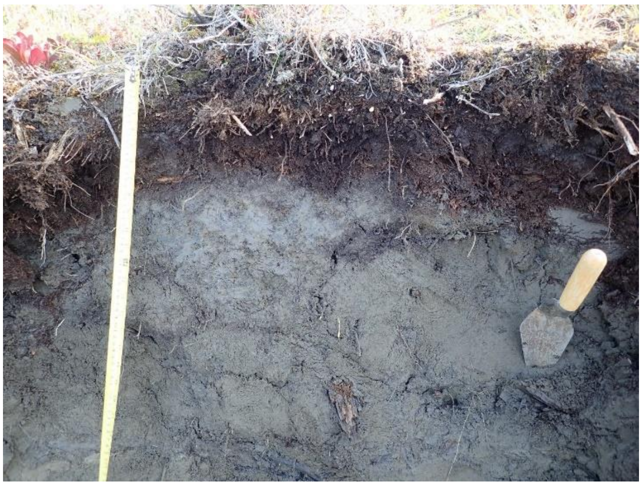

Supplementary Figure 42. **Close-up section through upper part of orthel.**

#12. Orthel-K-P1: spruce forest on loessal deposits

Coordinates: 63° 50' 25.9"N, 139°06' 42.8"W

Location: Little Blanche off Quartz Creek, Klondike goldfields, ~29 km SSE of Dawson City

Date of sampling: 23 August 2019

Geomorphic context: steep (~15–20°) hillslope leading down into valley bottom. Top of section ~15 m above valley floor

Vegetation: open spruce forest, willow shrubs, moss, Lab. tea, willow herb, lichen, crowberry

Organic soil: black organic layer (8cm), spruce needles, moss, horsetail; humic in lower part

Mineral soil: dark grey silt–sand (loessal), containing abundant rootlets. Loessal ('muck') deposit.

ALT: 30 cm beneath brown dry mossy organic layer, measured in vertical section

Ground ice: near-surface permafrost ice-rich, with well-developed lenticular cryostructure, abundant rootlets and common wood fragments; top of grey ice wedge at ~2m depth, below sampling profile

Sampling: vertical section produced by placer mining; samples 19-194-K-P1 to 19-210-K-P1

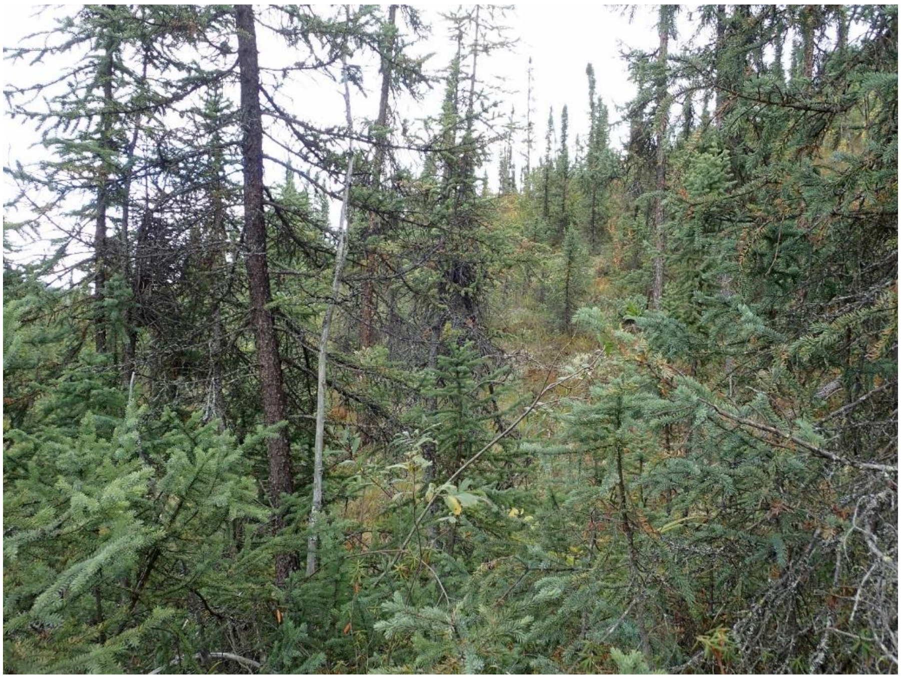

Supplementary Figure 43. **Spruce forest on hillside near sampling site.**

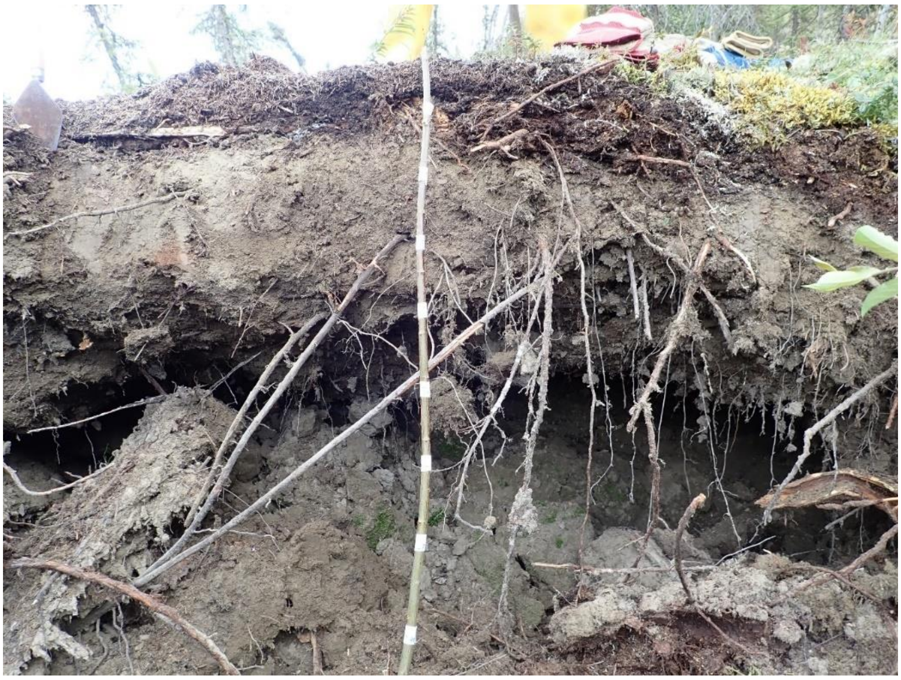

Supplementary Figure 44. **Vertical section through orthel.** White marks are spaced 10 cm apart on stick.

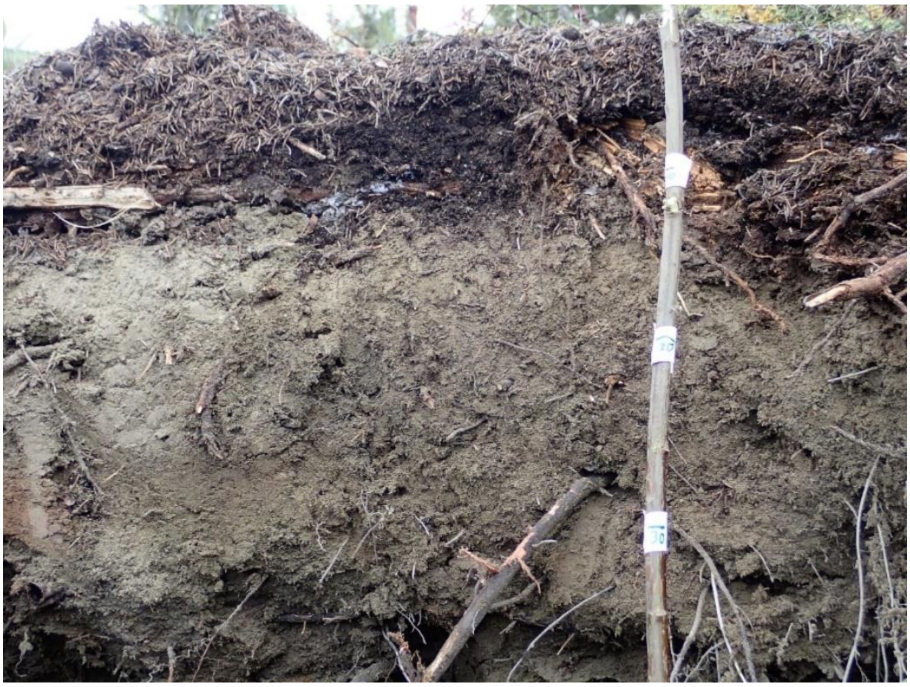

Supplementary Figure 45. **Close-up of upper part of orthel.** Numbers on white tape indicate depth (cm) below ground surface. Large soil sample 19-194-K-P1 from grey silt–sand (loessal) at 18–32 cm depth.

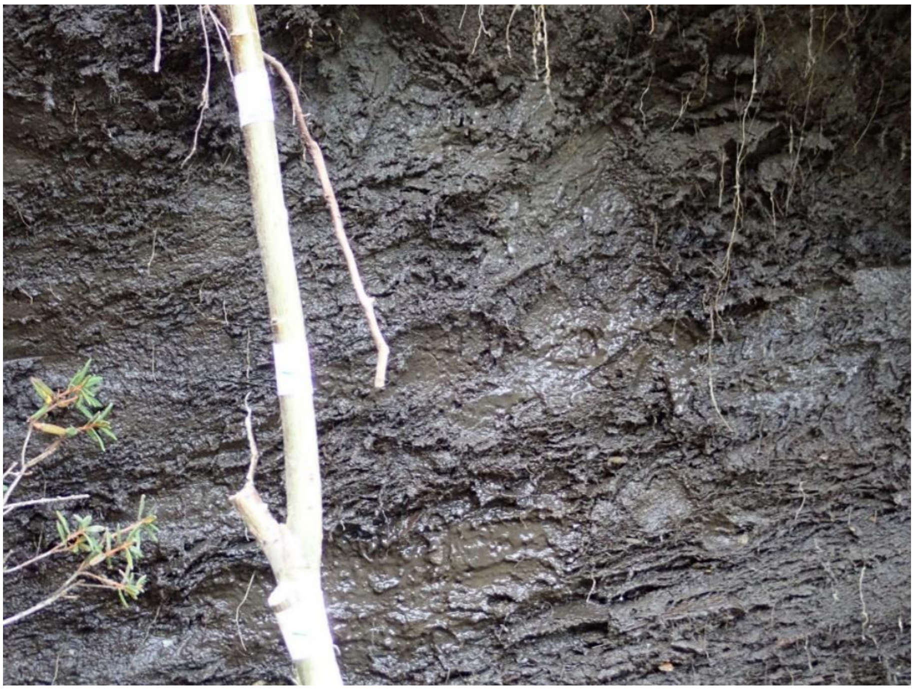

Supplementary Figure 46. **Close-up of lower part of orthel in near-surface permafrost.** Grey silt–sand is ice-rich (segregated ice). Abundant rootlets in mineral soil. Large soil sample 19-195-K-P1 from grey silt–sand (loessal) at 60–110 cm depth.

#13. Orthel-K-P2: spruce forest on loessal deposits

Coordinates: 63° 50' 30.9"N, 139°06' 53.1"W

Location: Little Blanche off Quartz Creek, Klondike goldfields, ~29 km SSE of Dawson City

Date of sampling: 23 August 2019

Geomorphic context: hillslope (~15–20°) leading down into valley bottom, ~200 m up valley of P1. Top of profile ~25 m above valley floor.

Vegetation: as at P1. Abundant green moss and lichen cover between spruce.

Organic soil: 20 cm thick, brown, dry, very mossy, fibric to mesic O horizon with sharp gently undulating lower contact above A horizon of dark grey silt

Mineral soil: dark grey silt (loessal) of Pleistocene age; part of an ice complex containing large syngenetic ice wedges

ALT: 35–45 cm, measured in vertical section

Ground ice: well-developed lenticular cryostructure; abundant tiny rootlets in near-surface permafrost.

Sampling: vertical section produced by placer mining; samples 19-211-K-P2 to 19-227-K-P2

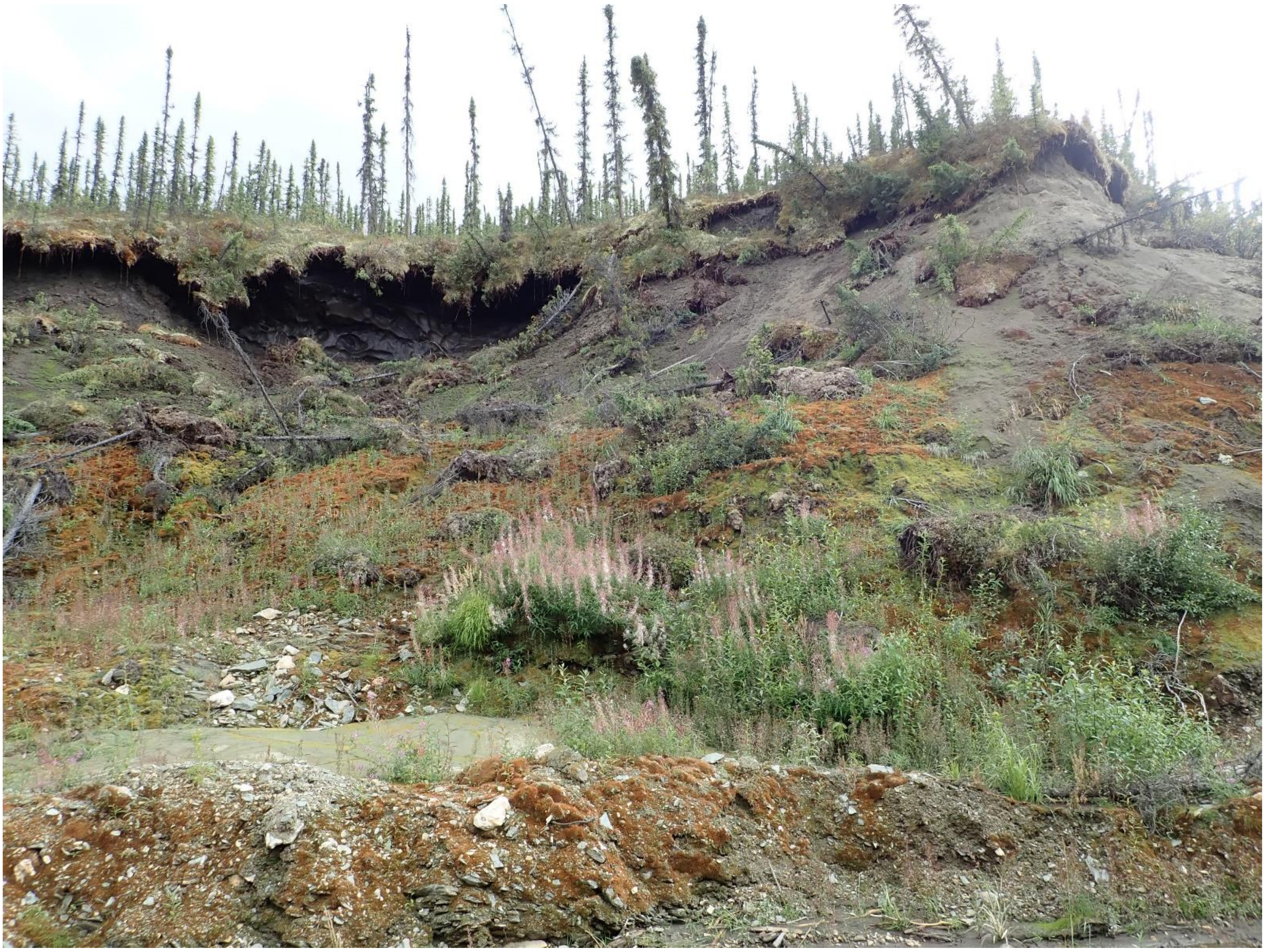

Supplementary Figure 47. **Spruce forest on hillside near sampling site.** Vegetation mat collapsing over top of headwall.

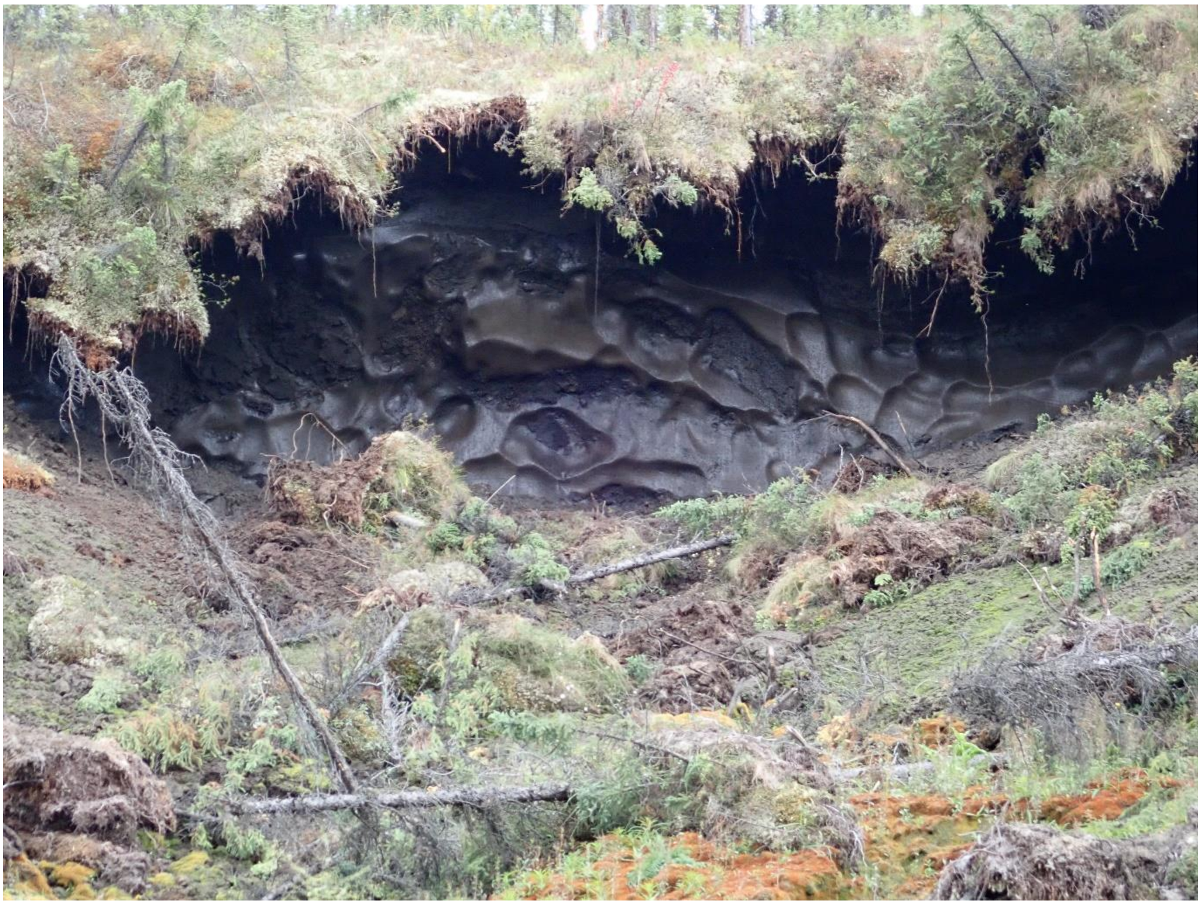

Supplementary Figure 48. **Ice-wedge ice with surface melt scallops.** Host material is dark grey ice-rich silt (loessal) of Pleistocene age.

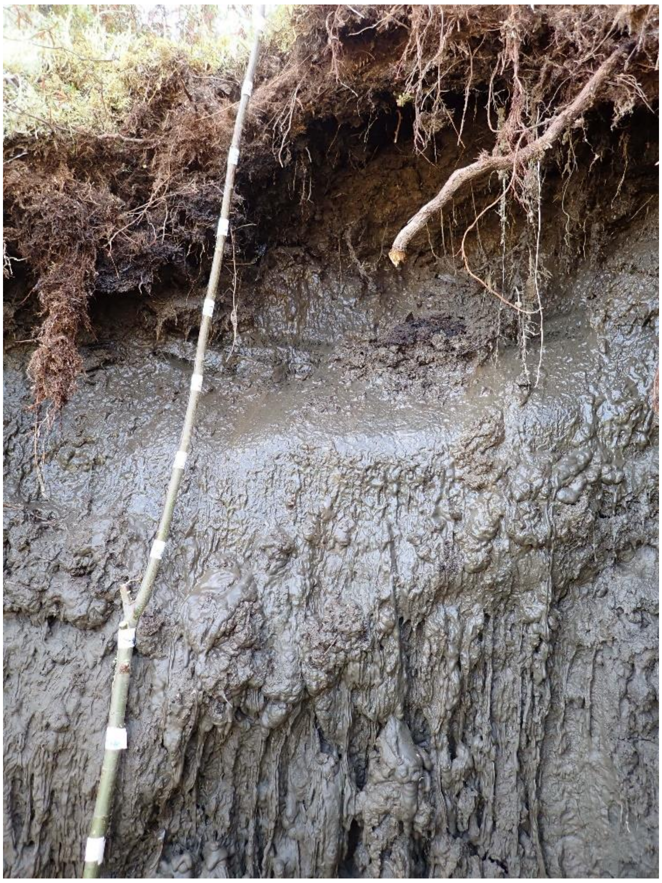

Supplementary Figure 49. **Vertical section through orthel**. White marks are spaced 10 cm apart on stick.

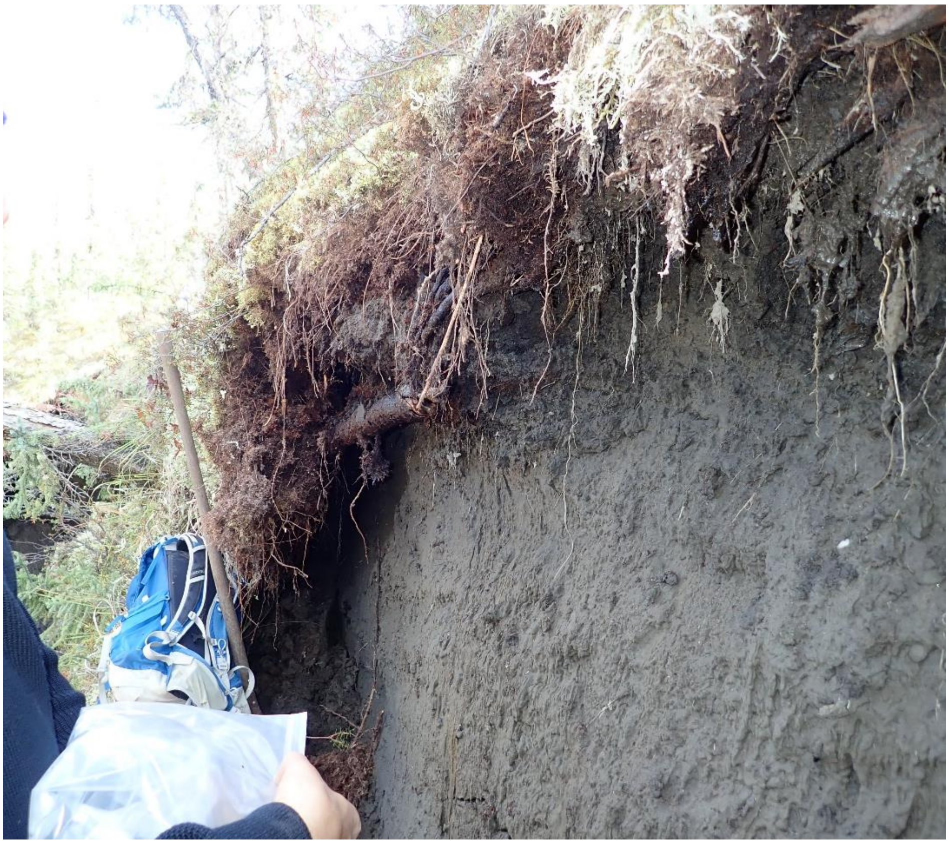

Supplementary Figure 50. **Close-up of upper part of orthel, showing organic layer above mineral soil.** Sample 19-211-K-P2 was from dark grey saturated silt at 20–35 cm depth.

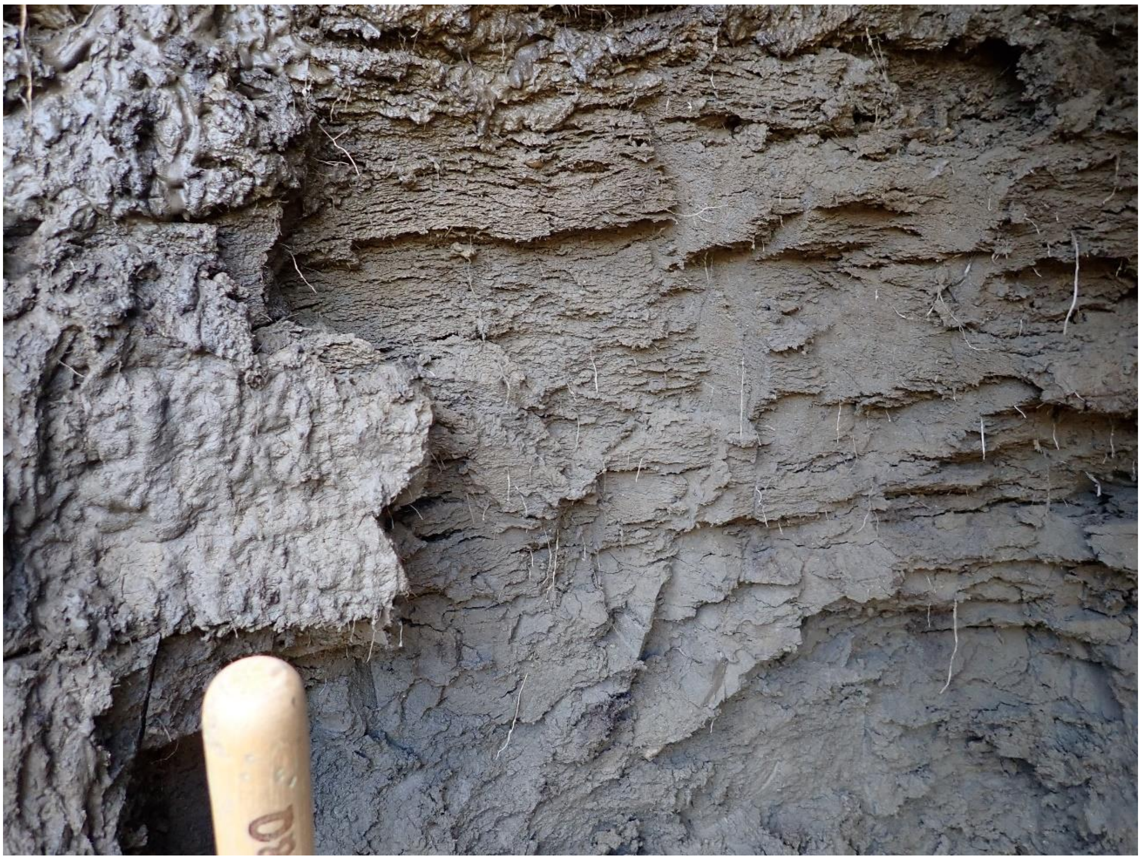

Supplementary Figure 51. **Close-up of lower part of orthel, showing dark grey ice-rich silt (loessal) of Pleistocene age.** Sample 19-212-K-P2 was from dark grey saturated silt at 120–140 cm depth. Horizontal fissures mark melt out of thin ice lenses of lenticular cryostructure. Note abundant small in situ rootlets, typical of yedoma deposits.

#14. Orthel- K-P3: shrubby vegetation on disturbed ground

Coordinates: 63° 55' 55.0"N, 138°54' 33.3"W

Location: Mint Gulch, Klondike goldfields, ~29 km southeast of Dawson City

Date of sampling: 24 August 2019

Geomorphic context: hillslope with irregular topography cover in shrubby vegetation near base of rectilinear hillslope covered in spruce forest. ⇨ Disturbed by placer mining activity several decades or more ago.

Vegetation: closed spruce forest with scattered aspen. Above P3 are very dense shrubs (willow, alder…) 1–3 m high, above grassy and mossy ground cover ⇨ young vegetation on disturbed site

Organic soil: mossy, fibric O horizon

Soil: heterogeneous in terms of texture and colour, with mineral-rich and organic-rich parts, forming lenses parallel to hillslope. ⇨ recent soil developed on mining spoil since gold rush in 1890s.

Subsoil: grey mineral silt, faint horizontal to subhorizontal strata (loessal); contains multiple buried palaeosols, Pleistocene age

ALT: 50 cm, measured in vertical section

Ground ice: not observed

Sampling: vertical section produced by placer mining; samples 19-228-K-P3 to 19-244-K-P3

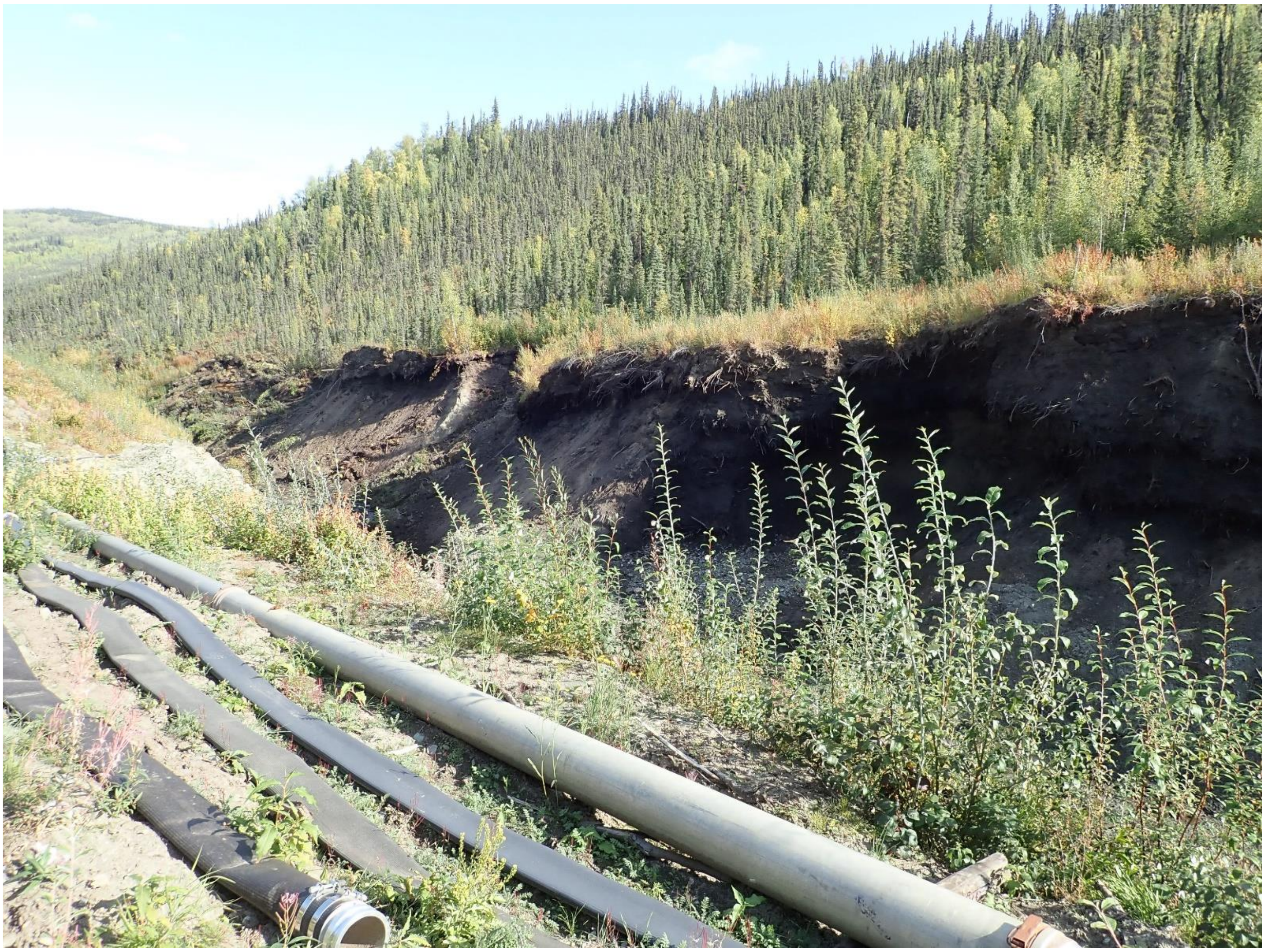

Supplementary Figure 52. **Shrubby vegetation and mounds (mining spoil) near P3.** Closed spruce forest on hillside in distance.

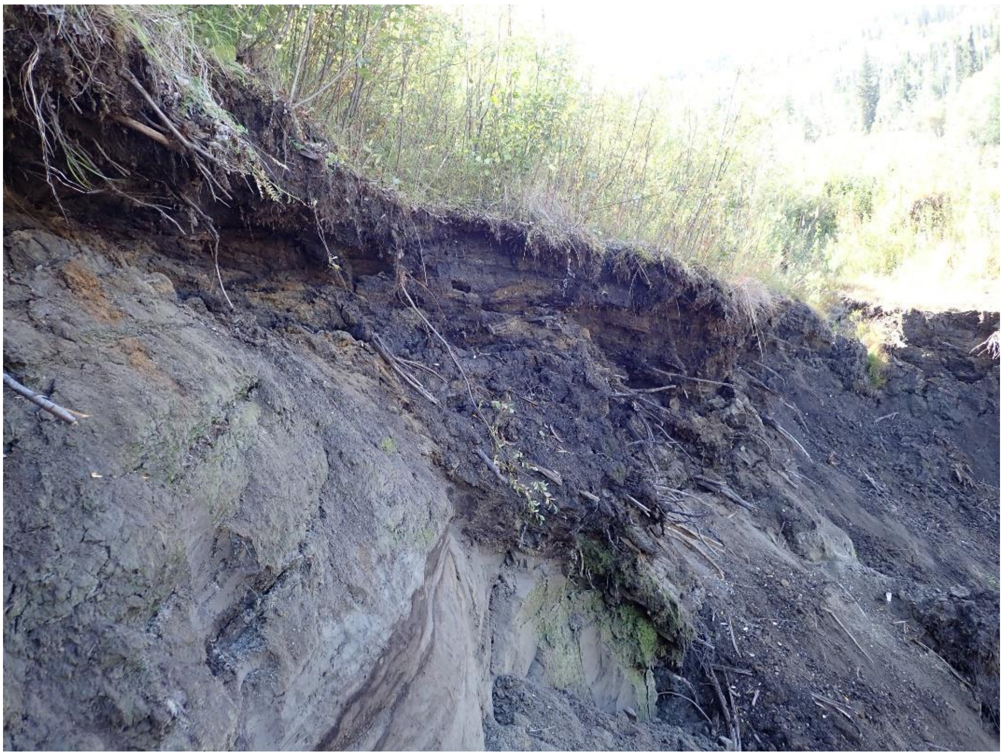

Supplementary Figure 53. **Scrubby vegetation on mining spoil near profile 3.**

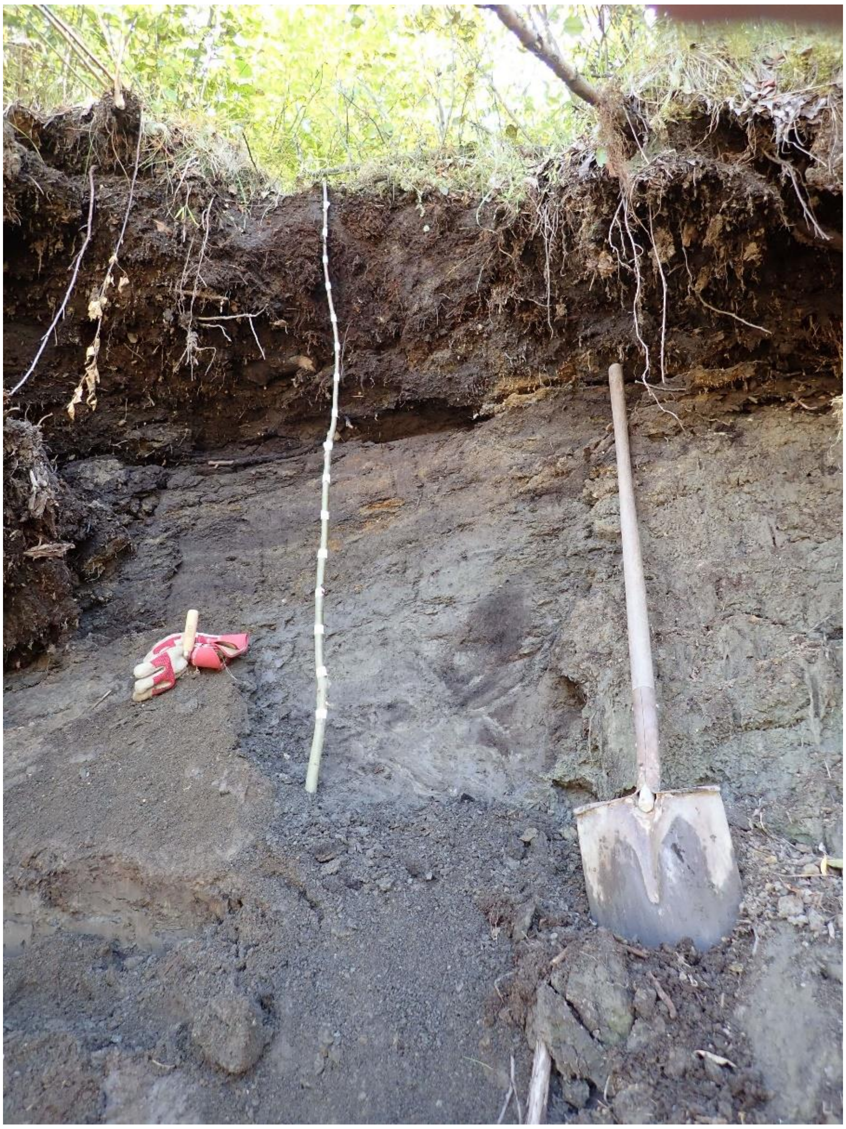

Supplementary Figure 54. **Vertical section through orthel.** White marks are spaced 10 cm apart on stick.

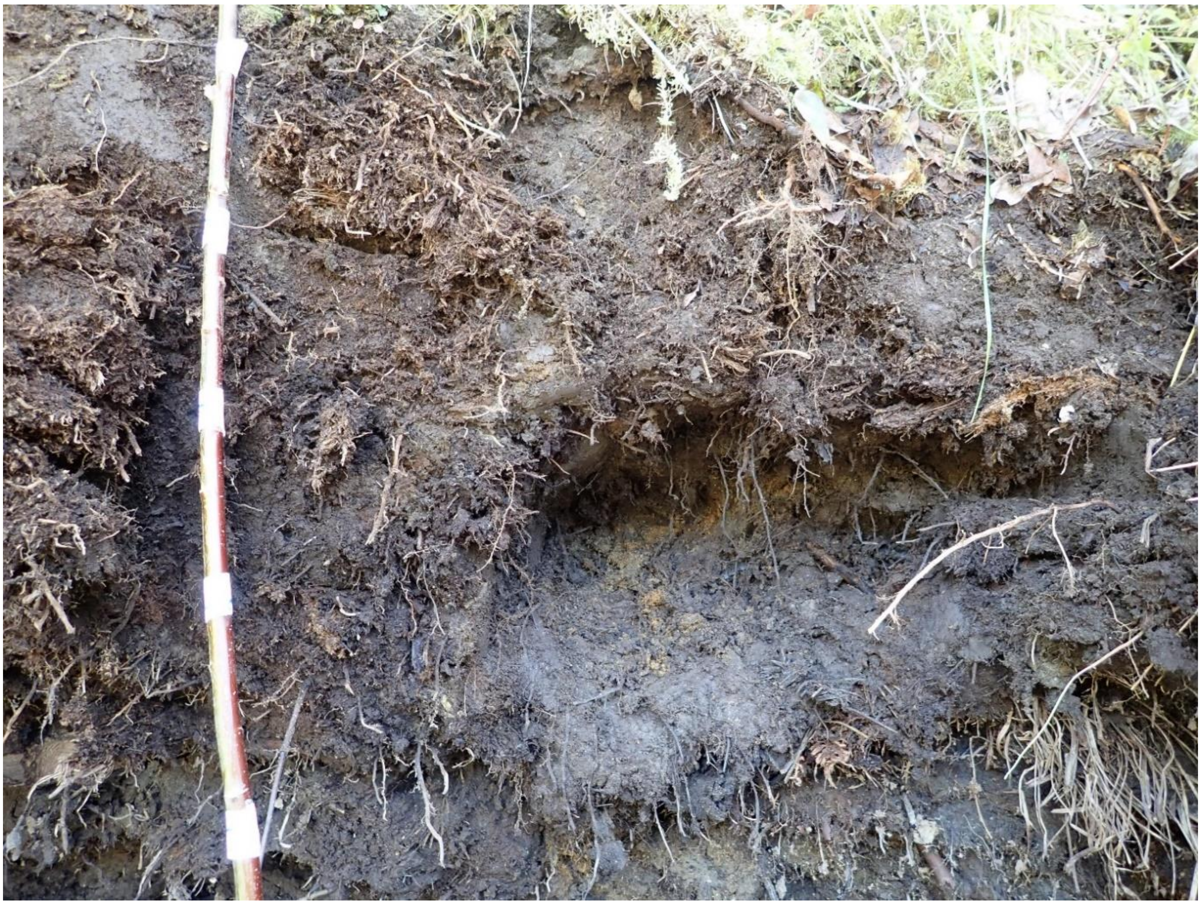

Supplementary Figure 55. **Vertical section through orthel.** White marks are spaced 10 cm apart on stick. Large soil sample 19-228-K-P3 collected from heterogeneous mineral–organic soil (mining spoil) at 20–50 cm depth (from large hole in centre).

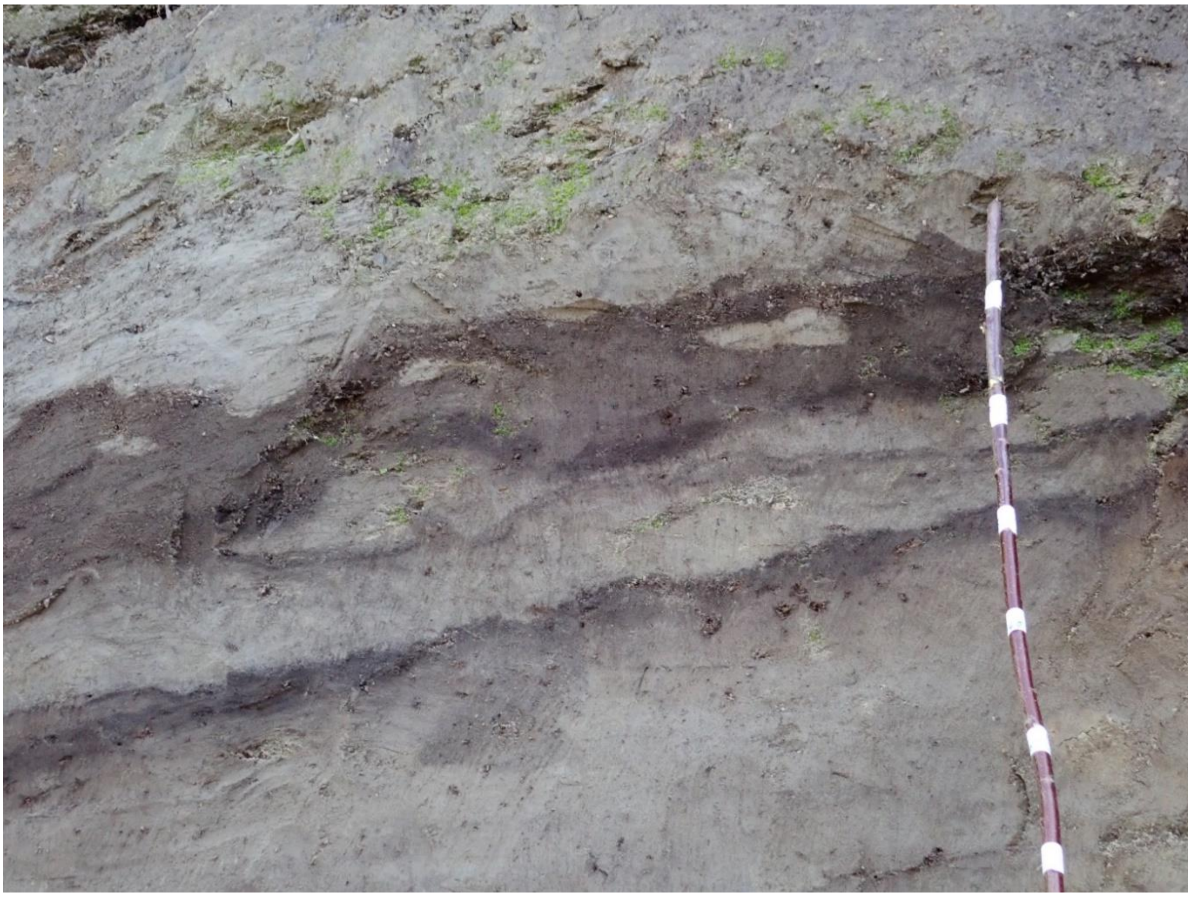

Supplementary Figure 56. **Stacked, buried palaeosols (black to dark brown) in mineral silt below base of profile 3.**

<u>CRB_S Turbel: hummocky ericaceous shrub tundra</u>

Coordinates: 67° 10' 52.0242"N, 135° 43' 34.4166"W

Location: north side of km 29 on the Dempster Highway

Date of sampling: 25 August 2020

Geomorphic context: hillslope truncated by secondary headwall on the eastern side of large retrogressive thaw slump (megaslump). Secondary slump developed between 2011 and 2013 as lobe of debris from main slump crept downslope, pinning stream against eastern valley slope

Vegetation: ericaceous shrub tundra

Mineral soil: brown, oxidised, fine-grained diamicton containing shale clasts, blocky structure (colluvium)

Active-layer thickness (ALT): not measured

Ground ice: Segregated ice lenses 2–10 mm thick, ataxitic to reticulate cryostructure overlies banded ice and sediment (ice-rich Pleistocene relict ice)

Sampling: vertical section of headwall; samples CRB_1, CRB_2 and CRB_3.

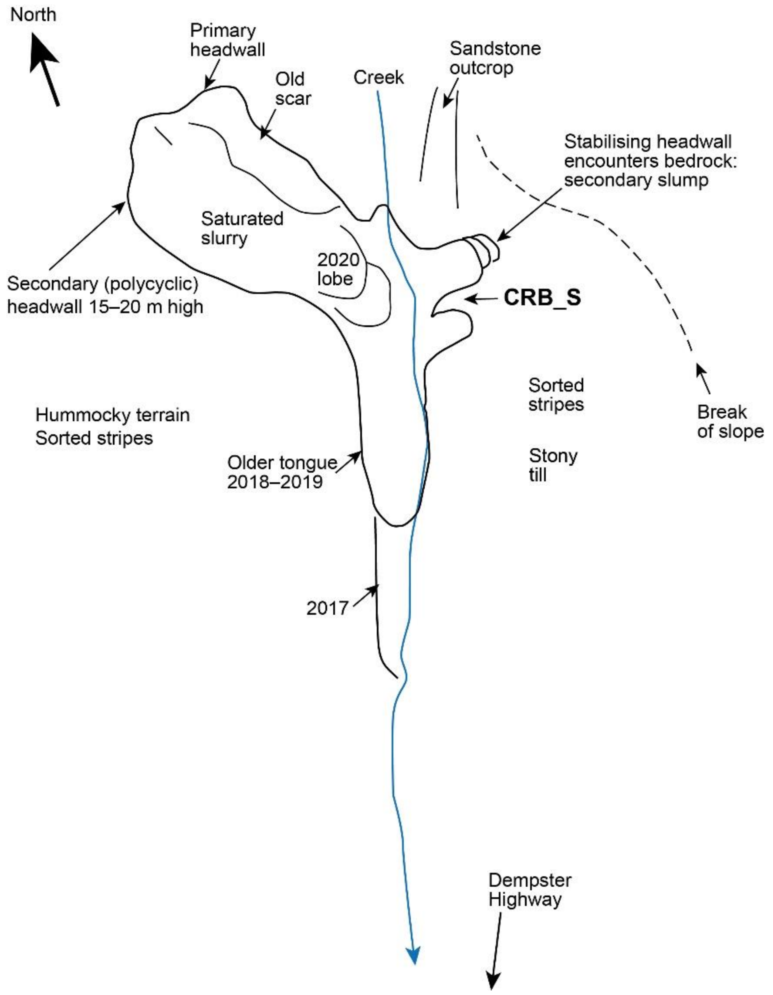


Supplementary Figure 57. **Sketch or CRB slump.** Sketch map (not to scale) of CRB slump showing location of secondary slump from where samples CRB_1, CRB_2 and CRB_3 were collected.

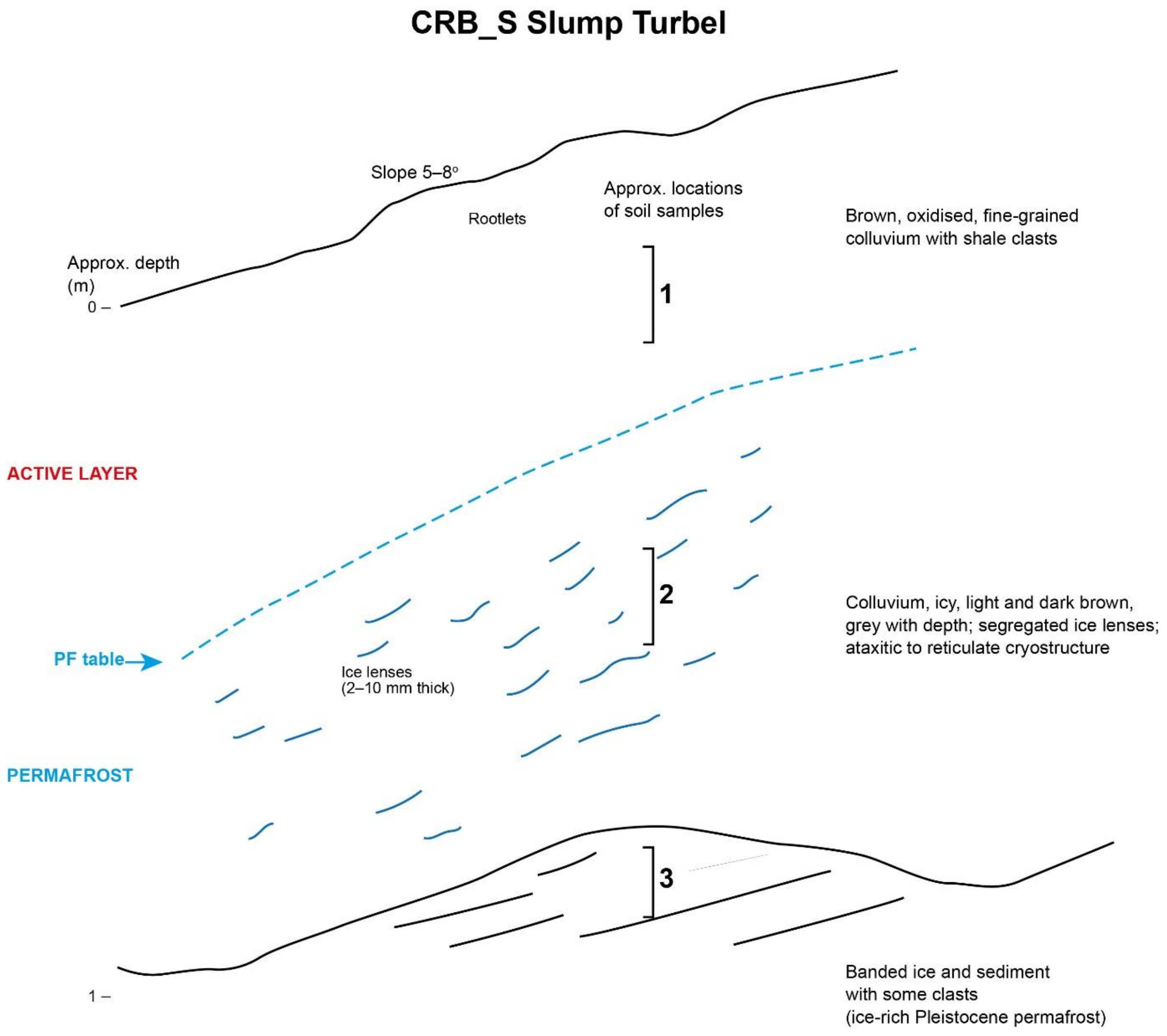


Supplementary Figure 58**. Schematic vertical section through CRB slump Turbel.** Schematic vertical section through CRB slump Turbel, showing approximate location of samples CRB_1, CRB_2 and CRB_3. Hand drawn originally by SVK, digital version made by JBM.

**Field descriptions of soil samples:**

CRB_S_1: active layer, blocky and mineral-rich, oxidised materials, mottled brown–grey; clasts of shale and sandstone, angular.

CRB_S_2: cryostructures are layered, lenticular, ice lenses 2–5 mm thick, dipping parallel to terrain surface (hillslope); shale/sandstone clasts, angular, up to 10 cm long.

CRB_S_3: cryostructures irregular, thick ice lenses, some massive cryostructure with ice lenses up to 10 cm thick; sediment-rich bands; rounded pebbles.

HUS Turbel: hummocky ericaceous shrub tundra

Coordinates: 69° 0' 53.7978"N, 133° 16' 45.951"W

Location: beside Inuvik–Tuktoyaktuk Highway, 49 km SSW of Tuktoyaktuk

Date of sampling: 23 September 2020

Geomorphic context: small retrogressive thaw slump beside small lake 2 km west of Husky Lakes

Vegetation: ericaceous shrub tundra

Organic soil: fibrous, ~15 cm thick

Mineral soil: diamicton (till), brownish in active layer, grey in permafrost, containing dark organic-rich streaks

Active-layer thickness (ALT): ~60 cm

Ground ice: segregated ice lenses 2–10 mm thick.

Sampling: vertical section in slump headwall; samples HUS_1, HUS_2 and HUS_3.

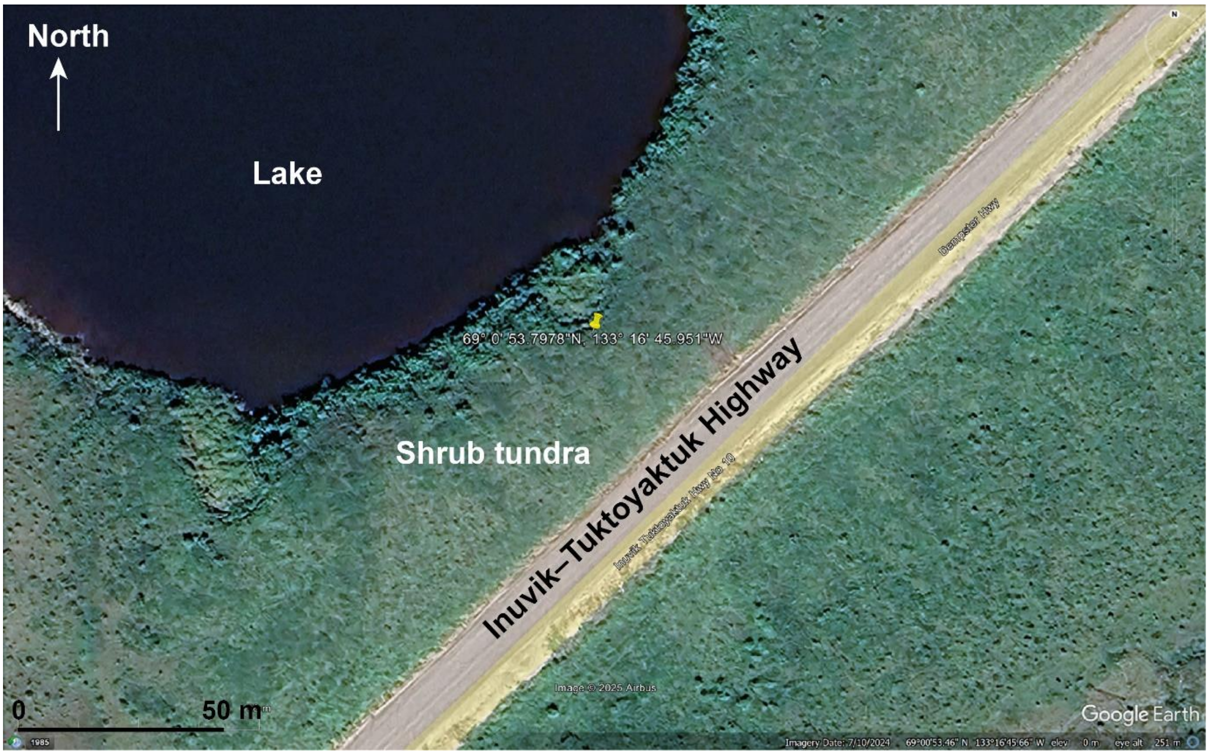


Supplementary Figure 59**. Satellite image of HUS slump location beside the Inuvik–Tuktoyaktuk Highway.** Yellow label shows sample site within active small thaw slump. Google Earth Imagery.

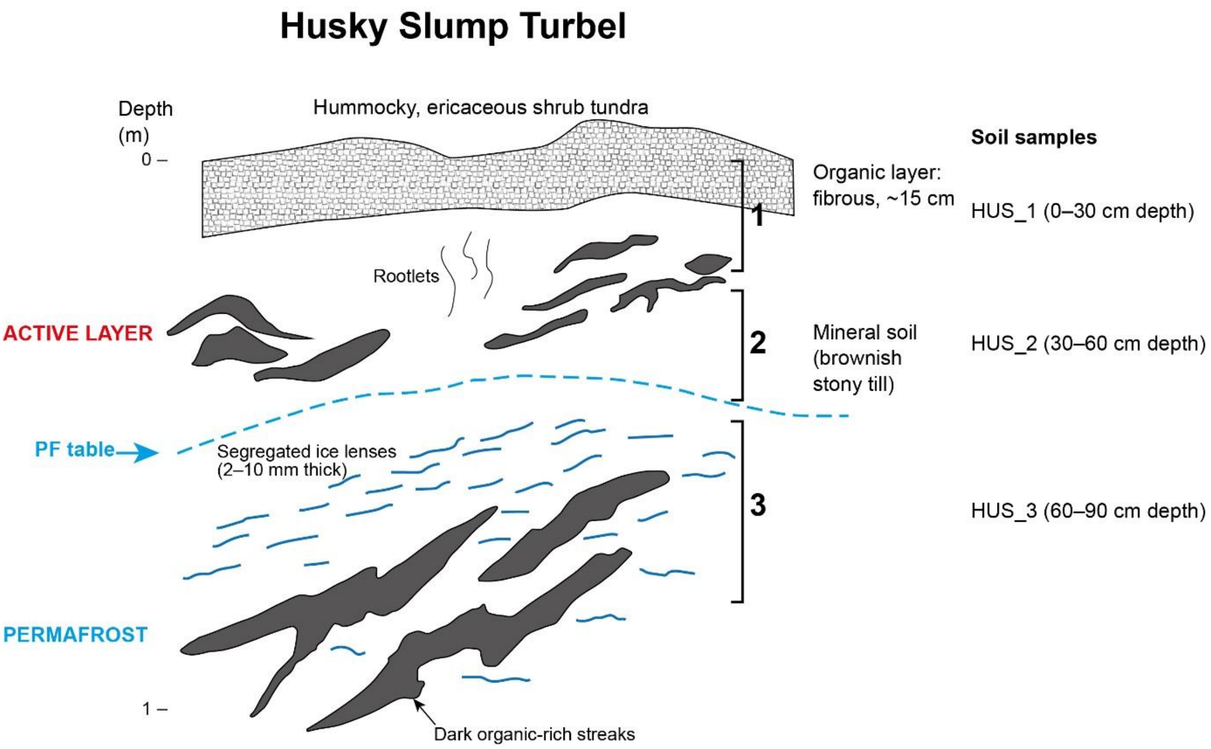


Supplementary Figure 60**. Schematic vertical section through HUS slump Turbel.** Schematic vertical section through HUS slump Turbel, showing location of samples HUS_1, HUS_2 and HUS_3. Hand drawn originally by SVK, digital version made by JBM.

**Field descriptions of soil samples:**

HUS_1: top of active layer, from lower organic layer to ~30 cm depth.

HUS_2: lower part of active layer; mineral soil with dark organic inclusions.

HUS_3: top 50 cm of permafrost; stony till.

# Methods

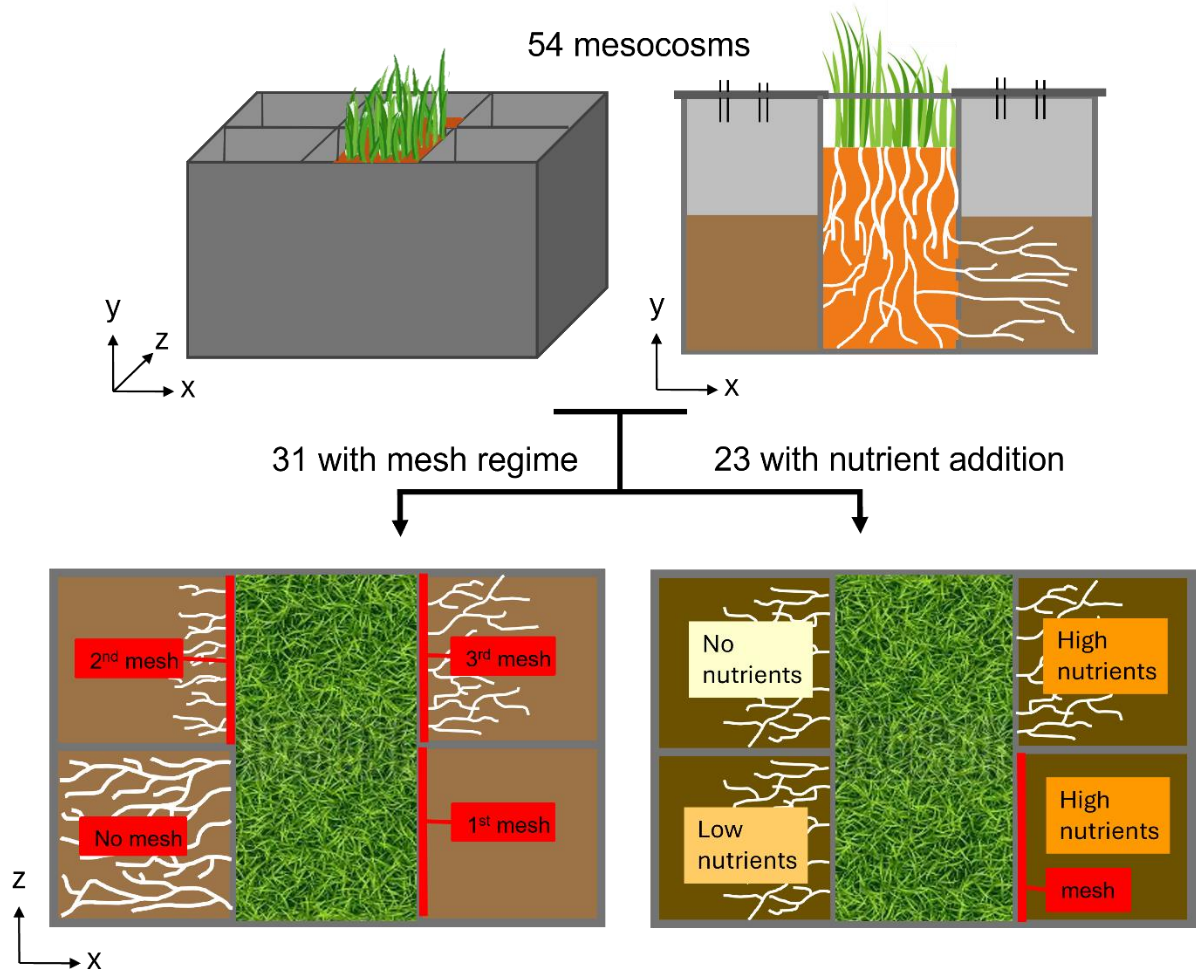


Supplementary Figure 61**. Schematic diagram of the mesocosm and experimental design.** 3D, side and plan views of the mesocosm and experimental design.
